# Influence of Co^2+^, Cu^2+^, Ni^2+^, Zn^2+^, and Ga^3+^ on the iron-based trimetallic layered double hydroxides for water oxidation†

**DOI:** 10.1039/d2ra01980a

**Published:** 2022-06-08

**Authors:** Jesus David Yong, Ricardo Valdez, Miguel Ángel Armenta, Noé Arjona, Georgina Pina-Luis, Amelia Olivas

**Affiliations:** Centro de Nanociencias y Nanotecnología – UNAM Km. 107 Carr. Tijuana-Ensenada C.P. 22860 Ensenada BC Mexico aolivas@cnyn.unam.mx; Tecnológico Nacional de México/Instituto Tecnológico de Tijuana, Centro de Graduados e Investigación en Química Blvd. Alberto Limón Padilla S/N, Mesa de Otay C.P. 22500 Tijuana BC Mexico ricardo.valdez@tectijuana.edu.mx; Universidad Estatal de Sonora, Departamento de Ingeniería en Geociencias Av. Niños Héroes, San Javier C.P. 84160 Magdalena de Kino Son Mexico; Centro de Investigación y Desarrollo Tecnológico en Electroquímica Parque Tecnológico Querétaro, Sanfandila C.P. 76703 Pedro Escobedo Qro Mexico

## Abstract

In this work, we synthesized five novel iron-based trimetallic layered double hydroxides (LDHs) by the urea-assisted co-precipitation method for the electrocatalytic water oxidation reaction (WOR). In particular, the synthesized electrocatalysts were labeled CoCuFe-LDH, ZnNiFe-LDH, ZnCoFe-LDH, ZnCuFe-LDH, and CoGaFe-LDH. The electrocatalysts were thoroughly characterized by means of Ultraviolet-visible spectroscopy (UV-Vis), N_2_ adsorption/desorption, and X-ray photoelectron spectroscopy (XPS). We analyzed the changes in the electronic structures, changes in the surface area, and the oxygen vacancies, respectively. X-ray diffraction (XRD) and transmission electron microscopy (TEM) showed that the materials had the hydrotalcite-like structure typical of LDHs. Electrochemical results indicated that the best electrocatalyst was the CoGaFe-LDH achieving an overpotential of 369.9 mV at 10 mA cm^−2^ and a Tafel slope of 64.8 mV dec^−1^ in alkaline conditions (KOH 1 M). Additionally, this material displayed a charge transfer resistance (*R*_ct_) of 30.1 Ω cm^2^. Electrochemical measurements indicated that the materials containing Zn^2+^ exhibit low kinetics; whilst materials with Co^2+^ or Ga^3+^ yield the best performances. The catalytic activity of the CoGaFe-LDH can be attributed to the decrease of the *R*_ct_ caused by electronic effects due to the addition of the Ga^3+^, lowering the thermodynamic barriers and thus enhancing the electron transfer. This work opens the door for a new approach to design efficient multimetallic catalysts based on the transition metals for WOR.

## Abstract 2

In this work, we synthesized five novel iron-based trimetallic layered double hydroxides (LDHs) by the urea-assisted co-precipitation method for the electrocatalytic water oxidation reaction (WOR).

## Graphical abstract

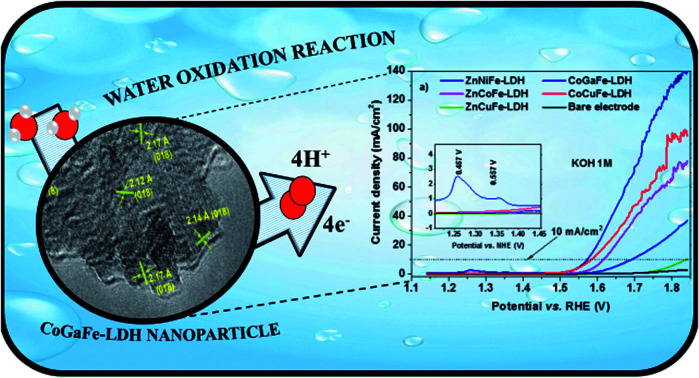

## Introduction

The demand for energy increases continuously as technology, quality of life, and population density increases; for this reason, it is necessary to find viable ways to produce renewable and environmentally friendly fuels.^1–3^ Molecular hydrogen is a great alternative to accomplish the energetic demand because of its high calorific value, which can be produced by electro- and photochemical water splitting.^1,3–5^ Water oxidation reaction (WOR) is crucial for the development of chemical energy generation through excitonic chemical conversion devices like artificial photosynthesis systems (PS).^2,6^ The challenge to achieve a scalable WOR is to overcome the high applied potential due to the nature of this multi-electron reaction:2H_2_O → O_2_ + 4H^+^ + 4e^−^which requires more than 1.23 V *vs.* SHE (standard hydrogen electrode). This high potential is required due to the multi-electronic nature of the reaction which requires to surpass an energetic barrier for each electron transfer.^7^

Precious metals like Ir or Ru and their oxides have been reported to be well-known active catalysts for the WOR in acid conditions, but these elements are expensive to be easily scalable.^8–10^ Moreover, several materials like perovskites, spinels, layered double hydroxides (LDHs), among others, have been reported for affordable WOR electrocatalysis.^4,11–14^ First-row transition metals like Fe, Co, Ni, Mn, Zn, and Cu have also been evaluated as cheaper catalysts and co-catalysts (more abundant on earth's surface) than precious metals for either photosystem of type 1 or 2.^4,5,7,15–17^

NiFe and CoFe LDHs have been engineered for enhancing the electrocatalytic activity and they are some of the most active catalysts for WOR based on transition metals.^18–20^ Nevertheless, few mechanisms or strategies have been proposed to modulate their electronic structure. Recently, Wang *et al.*, proposed that the incorporation of transition metals with 5d^*n*^ electronic configuration could modulate the electronic structure of materials based on 3d metals,^21^ however, they applied this only to the case of iridium which is a well-known catalyst for water splitting. Some studies indicate that trimetallic LDHs exhibit a higher performance than bimetallic LDHs. In particular, the electrocatalytic activity of CoNiFe-LDHs and CoFeV-LDHs of these materials were better than the bimetallic ones.^22^

One of the catalyst design strategies is to be guided by the existing ones and then varying the active species by doping, changing the synthesis method, and modifying the crystal structure or the catalyst morphology.^23^ The first parameter aims to modify the electronic structure of the catalysts by adding other atoms or ions. The second one refers to increase the amount of available active sites where the reaction will take place. The last one is seeking to change the energies of the active sites through modifying their molecular geometry or coordination.^23–25^

Lately, a novel strategy in the catalyst design is the incorporation and increasing of oxygen vacancies. In recent times, research groups have been demonstrated that the oxygen vacancies enhanced the water oxidation catalysis. For instance, Wang *et al.*, synthesized an exfoliated nitrogen-doped oxygen vacancy-rich NiFe-LDH for enhancing the WOR.^26^ Furthermore, Wang *et al.*, synthesized a LDH with oxygen vacancies grown on carbon nanotubes for efficient WOR.^27^

To the best of our knowledge, there are only a few reports related to the synthesis of catalysts based on chemical properties such as ionic radius, electron affinity, or energy of the orbitals involved in the electron transfer.^28,29^ Hence, it is imperative to develop new strategies for designing catalysts based on the concepts already mentioned.

Here, we propose a new strategy in the design of novel trimetallic LDHs based on iron by applying fundamental concepts like ionic radius, coordination number, spin state, and crystal field effects, relevant to improve the catalyst performance towards WOR. We synthesized a series of new iron-based trimetallic LDHs by the urea-assisted co-precipitation method and evaluated them in the electrocatalytic WOR. Moreover, we studied the UV-vis data, the specific surface area, and the XPS spectra of these materials to monitor the changes in their electronic structures, changes in the surface area, and the number of oxygen vacancies (O_v_) respectively, aiming remarkable improvement in water oxidation electrocatalysis.

## Experimental section

### Materials

All chemical reagents were analytical grade and supplied by Sigma Aldrich. They were used without further purification. Deionized water, Fe(NO_3_)_3_·9H_2_O (purity ≥ 99.95%), Co(NO_3_)_2_·6H_2_O (purity ≥ 98%), Ni(NO_3_)_2_·6H_2_O (purity ≥ 98.5%), Cu(NO_3_)_2_·3H_2_O(purity ≥ 98%), Zn(NO_3_)_2_·6H_2_O, and Ga(NO_3_)_3_·*x*H_2_O (purity ≥ 98%) were used in the LDHs synthesis. The main text of the article should appear here with headings as appropriate.

### Synthesis of trimetallic LDHs

The trimetallic LDHs were synthesized through a slightly modified urea-assisted co-precipitation method.^17^ Typically, in a two-neck flask 100 mmol of urea, 10 mmol of divalent metals, and 4.28 mmol of trivalent metals (trivalent metal molar fraction = 0.33) were dissolved in 100 mL of distilled water. The homogeneous solution was heated at 90 °C for 8 h with moderated magnetic stirring. The resulting solution had a pH of ∼9 and was aged for 13 h at 65 °C. Subsequently, the slurries (∼1.2 g) were recovered by decantation and then the products were washed with distilled water and centrifuged 3 times at 5000 rpm for 30 min. Finally, the synthesized LDH materials were dried in a Lindberg Blue M programmable oven at 100 °C for 16 h.

### Physicochemical characterization of LDHs

X-ray powder diffraction (XRD) patterns were recorded on a D2 PHASER diffractometer using Cu Kα (*λ* = 1.54056 Å) radiation. The analyses were carried out in a Bragg–Brentano configuration from 10 to 80° in 2*θ* with a step size 0.02° and time of step of 0.5 s, with an operating voltage and current of 40 kV and 30 mA. N_2_ adsorption/desorption measurements were performed using a Micromeritics model Tristar II equipment at −196 °C. The specific surface area was calculated by the Brunauer–Emmett–Teller (BET) method, and the pore volume and diameter through the Barrett, Joyner, and Halenda (BJH) method. The samples were degassed overnight to 110 °C before the measurements.

Fourier transform infrared (FT-IR) spectra were obtained by a Bruker Tensor 27 spectrometer from 350 to 4000 cm^−1^ for 50 scans with a spectral resolution of 6 cm^−1^. The FT-IR spectra were corrected to the blank (KBr), atmospheric noise, as well as shifted to the baseline. Thermogravimetric analyses (TGA) were carried out using a TA SDT-Q600 Instruments equipment. In a typical TGA analysis, ∼3 mg of catalyst were placed on alumina pans and heated from 30 to 830 °C using an airflow of 100 mL min^−1^ at a temperature ramp of 10 °C min^−1^.

Ultraviolet-visible (UV-vis) studies were carried out with an Agilent Technologies spectrophotometer (Cary 5000 UV-Vis-NIR) that uses a tungsten lamp as the visible and infrared light source and a deuterium lamp as the ultraviolet light source. Furthermore, the spectrophotometer counts with an integration sphere made with a reference material “spectralon”. UV-vis spectra were recorded from 200 to 1100 nm in the reflectance mode and corrected to a blank (MgO) and baseline.

X-ray photoelectron spectroscopy (XPS) measurements were performed in a SPECS PHI-548 spectrometer. The instrument is equipped with a monochromatic source consisting of an aluminum anode (Kα = 1486.6 eV, 300 W). Measurements were obtained for 4 cycles at an energy pass of 150 eV in the case of the survey and 16 cycles at an energy pass of 50 eV for high-resolution spectra. The XPS spectra were calibrated by adjusting the C_1s_ peak of the adventitious carbon for charging effects at a binding energy of 284.8 eV.

Energy-dispersive X-ray spectroscopy (EDS) analyses were performed through a JEOL JIB-4500 microscope. The electron gun was lanthanum hexaboride (LaB6) and operated at an accelerating voltage of 200 kV.

Transmission electron microscopy (TEM), selected area electron diffraction (SAED), and chemical mapping analyses were carried out using a JEOL JEM-2100F (STEM) microscope. This microscope is equipped with a field emission-Schottky type electron gun operated at an acceleration voltage of 200 kV. The samples were dispersed in isopropanol by sonication for at least 30 min, and then a drop of the solution was dropped onto a 300 mesh copper grid covered with an amorphous carbon film to further use.

### Electrochemical measurements

Electrochemical measurements were performed at room conditions using a three electrodes configuration; a glassy carbon electrode (GCE) was used as the working electrode, a saturated calomel electrode (SCE) as a reference electrode, and a graphite electrode as the counter electrode. The electrocatalytic evaluation was carried out in aqueous 1 M KOH solution (pH = 14) as electrolyte. From LDHs, catalytic inks were prepared, afterward, those inks were drop-casted on the GCEs, to form chemically-modified electrodes. Before the preparation of the composite electrodes, the GCEs were polished until mirror finish. The preparation of the catalytic inks consisted of mixing 3 mg of catalyst, a 77% V/V of 5 wt% isopropanol-Nafion® solution, and 0.5 mg of Vulcan® carbon per mg of catalyst, which was sonicated for at least 30 min. Then, the ink was added to the GCE and allowed to dry for at least 1 h at room conditions to give a final catalyst loading of ∼0.2 mg cm^−2^.

LSV measurements were performed from 100 mV to 800 mV at a sweep rate of 5 mV s^−1^. The potentials measured against the SCE were converted to the scale of the reversible hydrogen electrode (RHE) using the eqn (1):^30^

1 *E*_RHE_ = *E*_SCE_ + 0.241 + 0.059 × pH (V)

On the other hand, electrochemical cyclic voltammetry (CV) tests were carried out at ±50 mV from the open circuit potential to ensure that the measurement was recorded in the non-faradaic region.^8^ Furthermore, EIS analyses were performed at a potential of 1.64 V *vs.* RHE due to the reaction is occurring at this potential, with a disturbance frequency of 100 kHz to 1 Hz and an amplitude of 10 mV. We chose that potential value in EIS measurements to ensure the reaction takes place in all catalysts. The WOR stability test of the CoGaFe-LDH and CoCuFe-LDH were carried out by chronoamperometry at a potential to achieve 10 mA cm^−2^ using a GCE-rotating disk electrode (RDE) with a geometrical area of 0.0707 cm^2^ at 1600 rpm. LSV and CV measurements were obtained through a Digi-Ivy DY2300 potentiostat, the EIS curves were carried out using a CH Instruments 920D potentiostat, and chronoamperometry measurements were performed using a Metrohm Autolab potentiostat.

## Results and discussion

### Structural determination and chemical composition

**Fig. 1:**
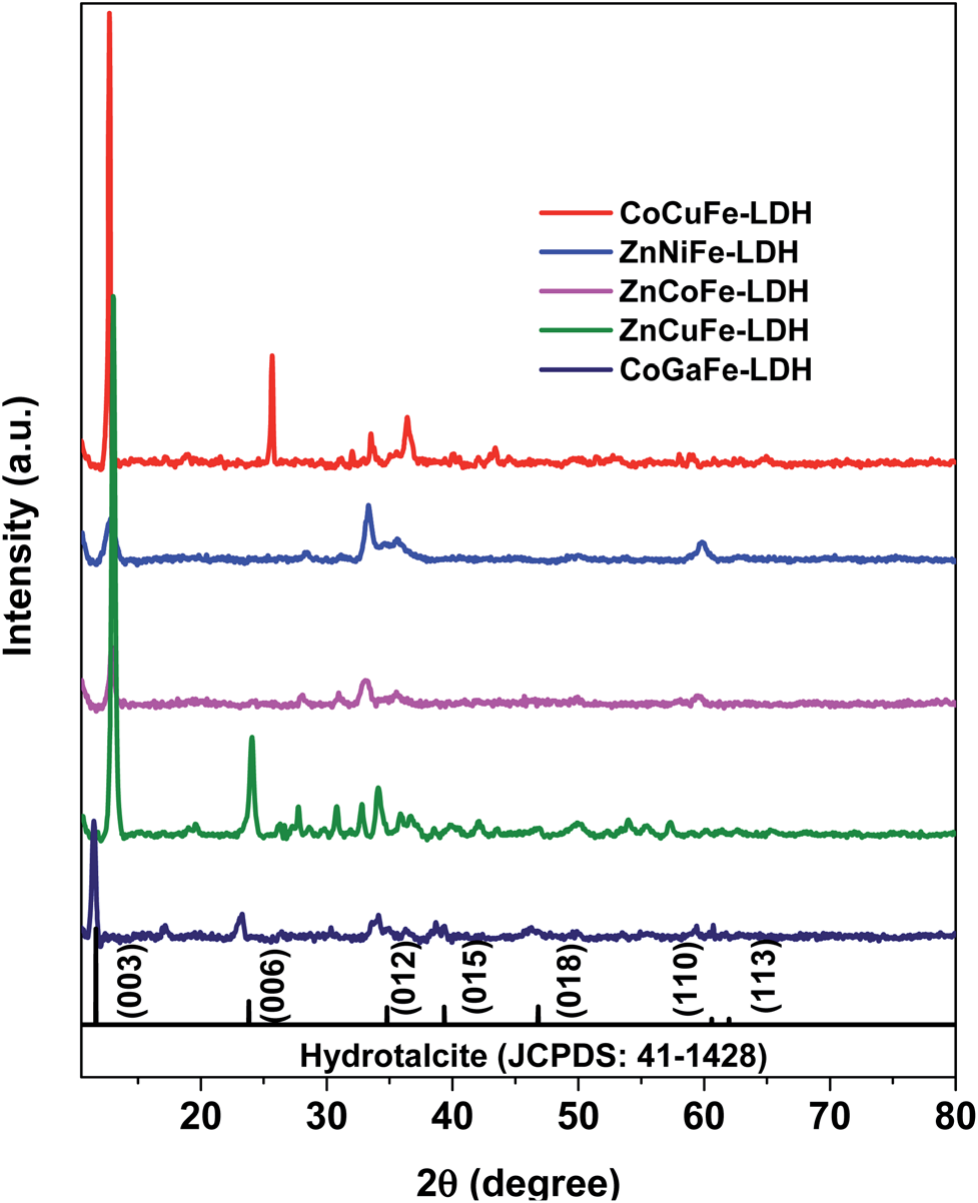
X-ray diffraction patterns corresponding to the synthesized LDHs.

The crystalline structures of the synthesized materials were determined through XRD (Fig. 1). X-ray patterns of all materials showed hydrotalcite characteristic diffractions (JCPDS: 41-1428) at 2*θ* of 13° (003), 24° (006), 34° (101), 36° (012), 39° (018), and 60° (110). The most intense peak (003) is shifted approximately 1° to the right in most of the patterns. 2*θ* shifts are attributed to the expansion or contraction of the crystal lattice, according to Bragg's law, due to structural defects or ionic substitutions within it. LDH patterns of the synthesized catalysts were compared with other crystallographic reference patterns that matched more closely than the hydrotalcite one (Table 1). The only material that showed a predominant phase other than hydrotalcite was the one labeled as CoCuFe-LDH which exhibited the presence of copper nitrate hydroxide (JCPDS: 45-0594). The lack of the peak corresponding to the (006) plane in ZnNiFe-LDH could be due to the molar ratio (>0.33) between cations causing the (006) plane intensity decreases.^31^ In the ZnCoFe-LDH, the low crystallinity is attributed to the concentration of trivalent metal compared to that of divalent metal (as will be seen later in the EDS analyses), which exceeds the concentration limit that an LDH can handle before deforming its structure.^32^

**Table 1:** Reference patterns (JCPDS) and textural properties of the LDHs synthesized

Material	JCPDS	BET area (m^2^ g^−1^)	Pore volume (cm^3^ g^−1^)	Pore size (nm)
CoCuFe-LDH	45-0594	36.5	0.0813	8.74
ZnNiFe-LDH	51-046333	92.9	0.1679	7.26
ZnCoFe-LDH	—	72.2	0.1667	8.63
ZnCuFe-LDH	38-0154	72.4	0.1121	5.64
CoGaFe-LDH	50-023534	91.7	0.1071	4.84

We observed type IV isotherms showing an H_3_ hysteresis loop in all the synthesized materials, which are characteristic of mesoporous materials (Fig. S1†). This type of isotherm and hysteresis loop do not present any limiting adsorption at high relative pressure, which is observed in aggregates of plate-like particles such as that of the LDHs.^33,34^

Table 1 also shows the textural properties of the LDHs synthesized. Here, we observed that the specific surface area decreases as: ZnNiFe-LDH > CoGaFe-LDH > ZnCuFe-LDH > ZnCoFe-LDH > CoCuFe-LDH. Moreover, we observed that the pore size is ranging from 4.84 to 8.74 nm which, again, agrees with the definition of mesoporous materials.^33^

**Fig. 2:**
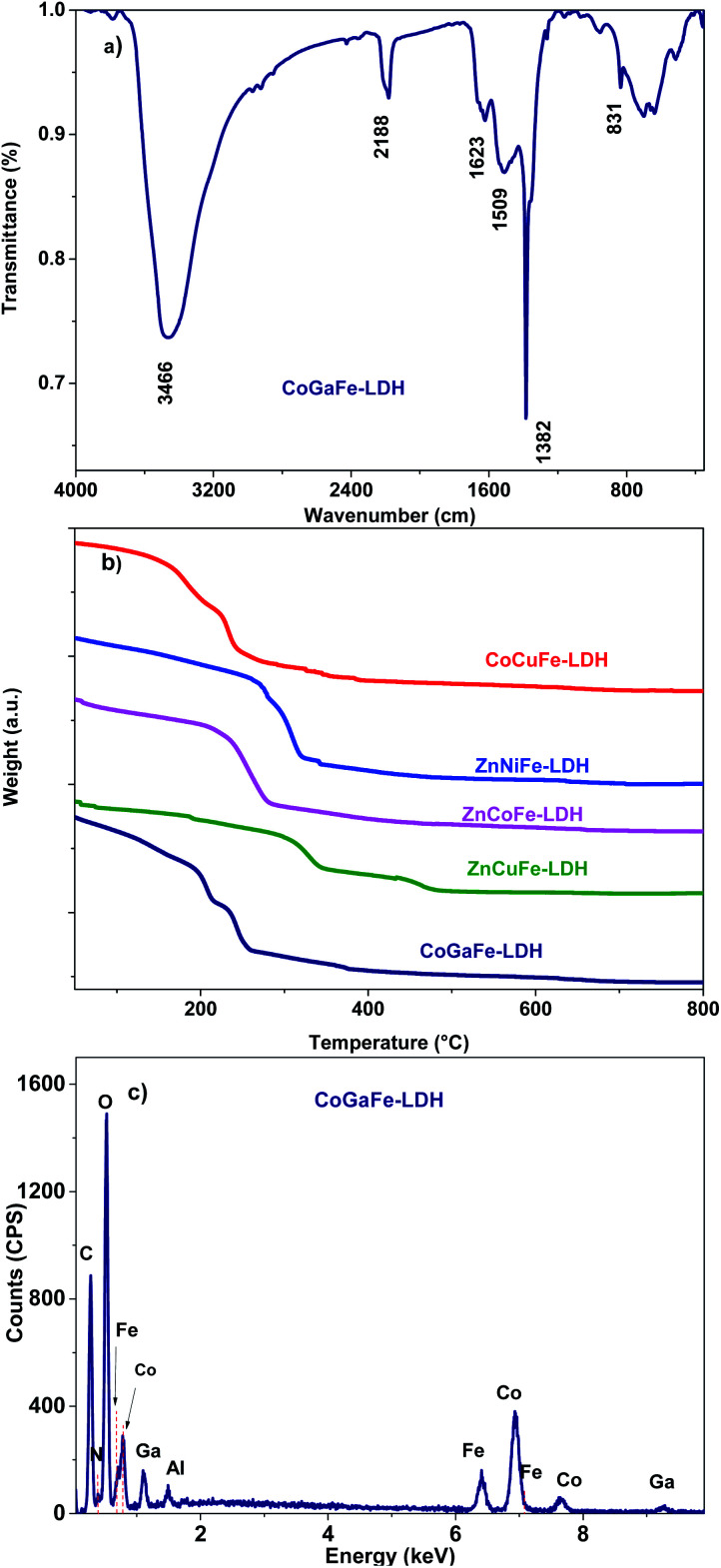
(a) FT-IR spectrum of CoGaFe-LDH. (b) Thermograms of the synthesized LDHs. (c) EDS spectrum of CoGaFe-LDH.

Interlaminar anions of the LDHs were identified by vibrational (FT-IR) spectroscopy. Fig. 2a displays the CoGaFe-LDH spectrum (see Fig. S2† for the FT-IR spectra of all the synthesized LDHs). The broad and intense bands observed between 3570–3390 cm^−1^ and the band centered around 1620 cm^−1^ are characteristic of the hydroxyl stretching modes on the LDH lattice and the bending modes of the interlaminar H_2_O molecules, respectively. The band around 2200 cm^−1^ is attributed to the presence of isocyanate anions from the decomposition of urea (eqn (2) and (3)), which decomposes at high temperatures to produce isocyanate and ammonium ions according to the following reaction:

2 CO(NH_2_)_2_ → NH_4_CNO

3 NH_4_CNO + 2H_2_O → (NH_4_)_2_CO_3_

The intense and sharp band at ∼1380 cm^−1^ is characteristic of nitrate ions (NO_3_^−^) that come from the metal precursors used during synthesis. The bands at 1050 (*ν*1) and 875 (*ν*2) cm^−1^ are ascribed to the deformation modes of CO_3_^2−^ ions. The *ν*3 vibration band at ∼1415 cm^−1^ is not obvious, because it may be hidden by other more intense bands such as the NO_3_^−^ one. However, the band at ∼1050 cm^−1^, which has been identified as characteristic of CO_3_^2−^ ions, was observed clearly in most of the LDH except for the CoGaFe, where the NO_3_^−^ seems to be the dominant species in the interlaminar region.^35^ These CO_3_^2−^ species come from the dissolution of atmospheric CO_2_ into the aqueous solution, as well as, from the urea decomposition reaction. The vibrations in the fingerprint area, in the wavenumbers < 800 cm^−1^, are ascribed to the distinct vibrations of the M–OH bonds.

Thermogravimetric measurements were performed to confirm the weight loss stages corresponding to the phase change from LDHs to mixed oxides (Fig. 2b). We observed that a dehydration process of the materials occurred in 2 stages (black arrows); the first one between ∼30 and 200 °C is due to weakly bound water, such as, water within the pores between LDH agglomerates. The second one is located between 200 and 350 °C and attributed to the bound water in the interlaminar region of LDHs.^32^ The weight losses at higher temperatures were attributed to the decomposition of the interlaminar anions (CO_3_^2−^ and NO_3_^−^) and to the dehydroxylation process that leads to the mixed oxide formation. Furthermore, we observed that the thermal stability of the materials follows the order: ZnCuFe-LDH > ZnNiFe-LDH > ZnCoFe-LDH > CoGaFe-LDH > CoCuFe-LDH, which indicates that the Zn^2+^ is playing a significant role as a thermal stabilizer while Co^2+^ has the opposite effect. The ZnCoFe-LDH thermogram does not show the typical two-step weight losses as the other materials, which is in agreement with the poor crystallinity observed in the diffractogram of this mate-rial. The EDS spectrum of the CoGaFe-LDH material is shown in Fig. 2c. We confirmed through EDS analyses that the cations were incorporated into the LDH as observed in Fig. 2c and S3.† In the spectra we noted a small peak of N, this peak is due to the presence of the interlaminar NO_3_^−^ anions. The low crystallinity of the ZnCoFe-LDH displayed in the diffractogram is due to the greater amount of Fe compared to that of Zn and Co, as shown in Table S2 and S3.† It is well-known that the LDHs allow only a 0.33 molar fraction of trivalent cations in comparison to all other cations present in a given LDH.^36^

**Fig. 3:**
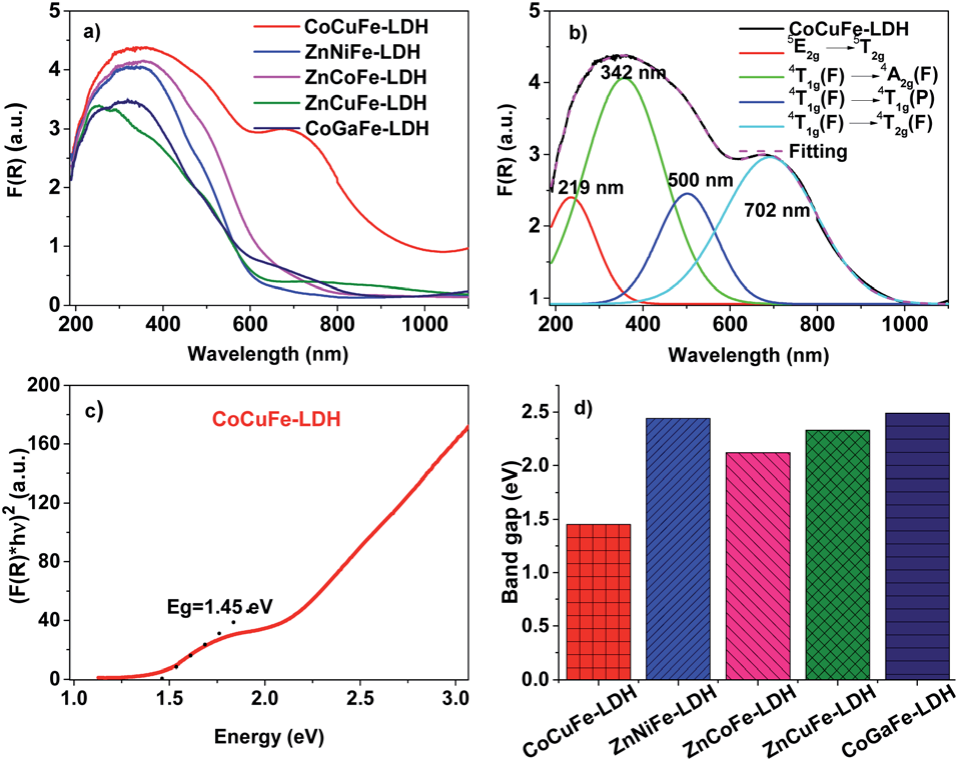
(a) UV-vis spectra of the LDHs. (b) UV-vis spectrum of CoCuFe-LDH and its electronic transitions. (c) Tauc plot of CoCuFe-LDH. (d) Band gaps of the LDHs.

UV-vis spectra of the synthesized LDHs are shown in Fig. 3a. We noted that all materials have a broad band from ∼200 to 570 nm, which is ascribed to several transitions that depend on the metal composition of the catalyst (Fig. S4†).^37^ In the Co^2+^ containing materials, the band at higher energy is attributed to ^4^T_1g_(F) → ^4^A_2g_(F) whereas the band at lower energy is assigned to ^4^T_1g_(F) → ^4^T_1g_(P) transitions.^38^ The CoCuFe-LDH material (Fig. 3b) has a wide absorption band ranging from 570 to 760 nm wavelengths with maximum absorption at ∼710 nm, this band is attributed to the ^4^T_1g_(F) → ^4^T_1g_(P) transition of Co^2+^.^37^ Additionally, these 3 bands are observed in all Co^2+^ containing materials as expected within a UV-vis spectrum, for a weak or moderately weak field octahedral material.^38^ The bands in the spectra should be shifted to higher or lower wavelengths if there are any metal cation that can shift the bands through an additional contribution from a transition to nearby energies, such as Cu^2+^ in the case of CoCuFe-LDH. In this case, we observed that the bands assigned to the Co^2+^ species shifted to lower energies because of the Cu^2+^ (^5^E_2g_ → ^5^T_2g_) transition. This displacement toward lower energies could be due to the coulombic repulsion between electronic shields of Cu^2+^ and Co^2+^, which would make the electrons in these atoms have additional energy, consequently, further transitions would need less energy than those that do not have this repulsion effect. In the ZnNiFe-LDH spectrum showed in the Fig. S4,† we can observe 3 bands attributed to the Ni^2+^ electronic transitions ^3^A_2g_(F) → ^3^T_2g_(F), ^3^A_2g_(F) → ^3^T_1g_(F), and ^3^A_2g_(F) → ^3^T_2g_(P) in octahedral sites located from lower to higher wavelengths.^38^ Metals with d^5^ or d^10^ configuration do not show electronic transitions in weak or moderately weak fields (such as Fe^3+^ or Zn^2+^). For this reason, there are no bands assigned to Zn^2+^ or Fe^3+^ species. However, there are forbidden transitions by symmetry that can be “relaxed” and shoulders could appear due to these processes of violation or “relaxation” of the selection rules.^38^

The complexity of UV-vis spectra, as in the case of ZnCuFe-LDH, may be due to effects such as Jahn–Teller type distortions (J–T) due to having cations with d^5^, d^9^, and d^10^ electronic configuration (Fe^3+^, Cu^+^, and Zn^2+^, respectively) within these materials. The first two electronic configurations are prone to octahedral distortions of this type (J–T), which would affect the crystallinity of the material. These observations are in agreement with the presence of other phases found in the CoCuFe-LDH material, as seen in XRD analyses. Fig. 3c shows the Tauc plot of the CoCuFe-LDH considering that the material presents a direct and allowed transition (see Fig. S5†).^39,40^ Besides, we observed that the curve of Fig. 3c has two absorption edges, which are attributed to different phases or different particle sizes, such as the CoCuFe-LDH. As seen in Fig. 3d, the Eg values from 1.45 to 2.49 eV indicate that the materials could have a potential application in semiconductor devices such as solar cells or in photocatalysis and photo-electrocatalysis.

**Fig. 4:**
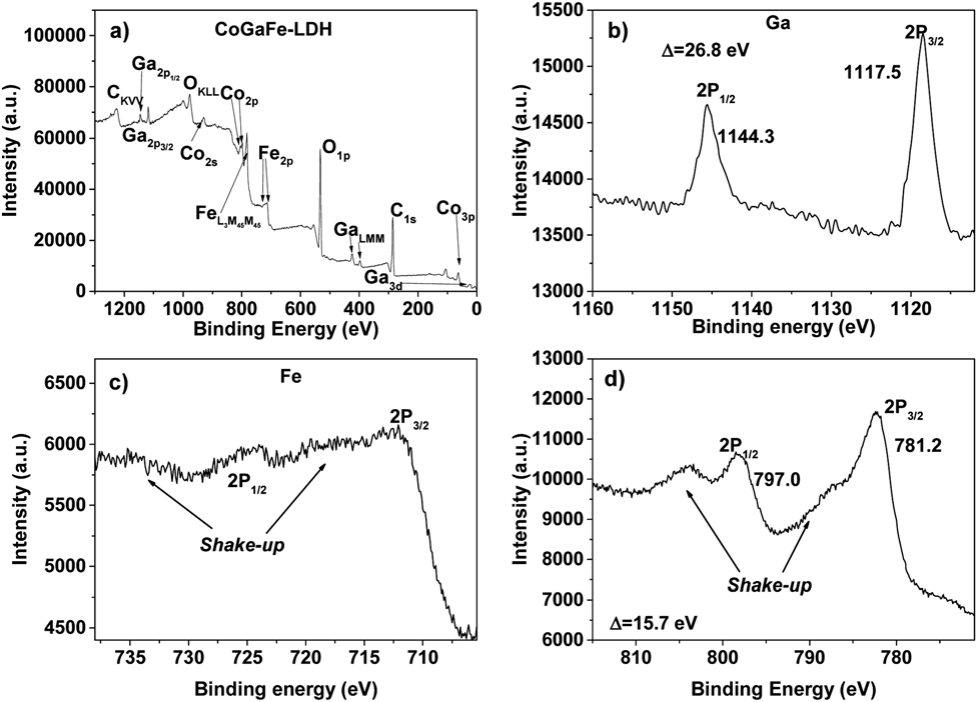
XPS spectra of CoGaFe-LDH. (a) Survey spectrum of CoGaFe-LDH. (b) Ga high-resolution spectrum. (c) Fe high-resolution spectrum. (d) Co high-resolution spectrum.

The chemical state and composition on the surface of the LDHs were determined by XPS. In Fig. 4 are shown the XPS spectra of CoGaFe-LDH. Through the survey spectrum (Fig. 4a), the presence of the elements previously presented by EDS was confirmed, which were consistent with each other. The most intense photoemission peaks at 284.8 and 532.2 eV corresponding to the C_1s_ and O_1s_ peaks, respectively, as well as the photoemission lines Fe_2p_ and Auger Fe_LMM_ at 711 and 781 eV, respectively, were identified in all materials (Fig. S6–S9†).

The main lines of photoemission and Auger peaks for the other metal components of the materials like Cu, Co, Ni, Zn, and Ga were identified in Fig. 4 and S6–S9† (Cu_3s_, Cu_3p_, Cu_LMM_, Co_2s_, Co_2p_, Ni_2p_, Ni_LMM_, Zn_3p_, Zn_LMM_, *etc.*). We identified the 2p_3/2_ peak and determined the spin–orbit splitting through the high-resolution spectra for the metal components of the samples presented in Fig. 4b–d, and in Fig. S6–S9.† The high-resolution spectra were meticulously analyzed to discard the presence of other chemical species.

Table S4† shows the position of the photoemission 2p_3/2_ peak in all materials. According to the literature, the Fe 2p_3/2_ peak located at 711.4 eV and the presence of two shake-up satellite peaks in all synthesized electrocatalysts suggest the presence of Fe^3+^ and Fe–OH bonding which appears in higher binding energies than Fe–O bond.^41,42^ Also, it is well-known that Cu_2_O appears at lower binding energies than 933 eV, instead, CuO appears shifted to higher binding energies.^42,43^ Also, 2p_3/2_ Cu peak in Cu(OH)_2_ is shifted to higher energies around 1.4 eV with respect to CuO.^41^ The values of 935.1 and 935.2 eV in Cu 2p_3/2_ peak of CoCuFe-LDH and ZnCuFe-LDH, respectively, suggest the presence of Cu^2+^ and the chemical species are bound with the OH^−^ instead O^2−^ anions. In Table S5† are presented the spin–orbit splitting values of the 2p doublets of all LDHs. Similarly, we carried out the same XPS analyses to determine the chemical state of all synthesized LDHs that turned out to be: Co^2+^, Ni^2+^, Zn^2+^, and Ga^3+^.^44–46^ These chemical species were those we expected according to the synthesis procedure and UV-vis analyses. Small differences between binding energies for the same species in different LDHs are attributed to different chemical environments. Table 2 shows the surface atomic percentages of the LDHs obtained by quantitative analysis. These results agree with those obtained by EDS.

**Table 2:** Surface atomic percentages in the materials synthesized

Material	%Ni	%Co	%Ga	%Cu	%Fe	%Zn
CoCuFe-LDH	—	37.0	—	14.8	48.2	—
ZnNiFe-LDH	42.8	—	—	—	34.9	22.3
ZnCoFe-LDH	—	38.7	—	—	45.6	15.8
ZnCuFe-LDH	—	—	—	31.5	39.3	29.2
CoGaFe-LDH	—	61.2	7.4	—	31.4	—

**Fig. 5:**
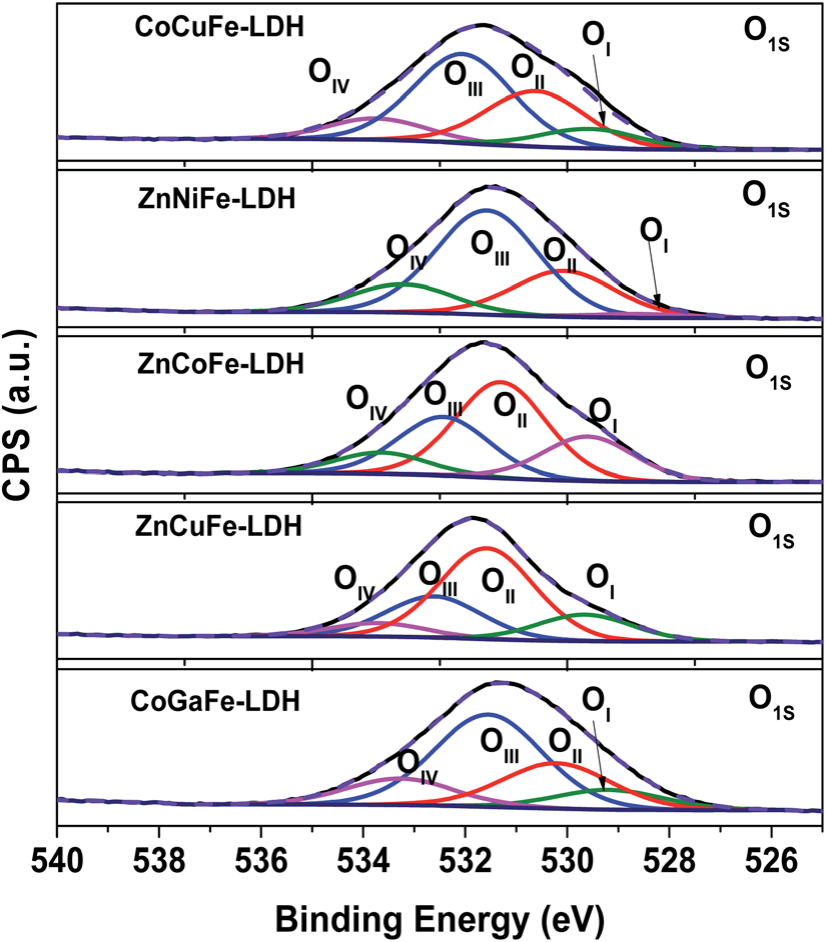
O_1s_ XPS high-resolution deconvolved spectra of the materials synthesized.

Fig. 5 shows the O_1s_ high-resolution XPS spectra of the materials synthesized. Through the O_1s_ photoemission peak deconvolution, we observed 4 different O-bonded species for all materials, these peaks come from the oxygen atoms bonded to the cations of the LDHs (O_I_), the oxygen atoms bonded to the metal in the vicinity of an oxygen vacancy (O_II_), the oxygen belonging to the hydroxyl groups or the surface adsorbed oxygen (O_III_), and the oxygen belonging to the water molecules (O_IV_) ranging from lowest to highest binding energies.^26,27^

Table 3 displays the O_I_, O_II_, O_III_, and O_IV_ percentage of the materials synthesized. From here, we determined that the amount of oxygen vacancies in the materials follows the trend: ZnCoFe-LDH > CoCuFe-LDH > CoGaFe-LDH > ZnNiFe-LDH > ZnCuFe-LDH. The last four materials have a similar concentration of vacancies whereas the ZnCoFe-LDH presented around 15% more oxygen vacancies. In addition, Table S6† shows the position of the oxygen bands of the O_1s_ deconvolved XPS spectra of the materials synthesized. The oxygen O_I_, O_II_, O_III_, and O_IV_ are located between 528.7–529.6 eV, 530.1–531.6 eV, 531.6–532.6 eV, and 533.2–533.7 eV, respectively. These differences arise from the different chemical compositions and environments in the materials.

**Table 3:** O_I_, O_II_, O_III_, and O_IV_ percentage on the synthesized materials

Material	O_I_ (%)	O_II_ (%)	O_III_ (%)	O_IV_ (%)
CoCuFe-LDH	10.64	29.98	47.86	11.52
ZnNiFe-LDH	10.68	24.71	50.22	14.39
ZnCoFe-LDH	20.08	43.65	26.69	9.58
ZnCuFe-LDH	15.35	23.95	52.88	7.83
CoGaFe-LDH	2.19	25.11	57.33	15.37

### Morphological characterization

**Fig. 6:**
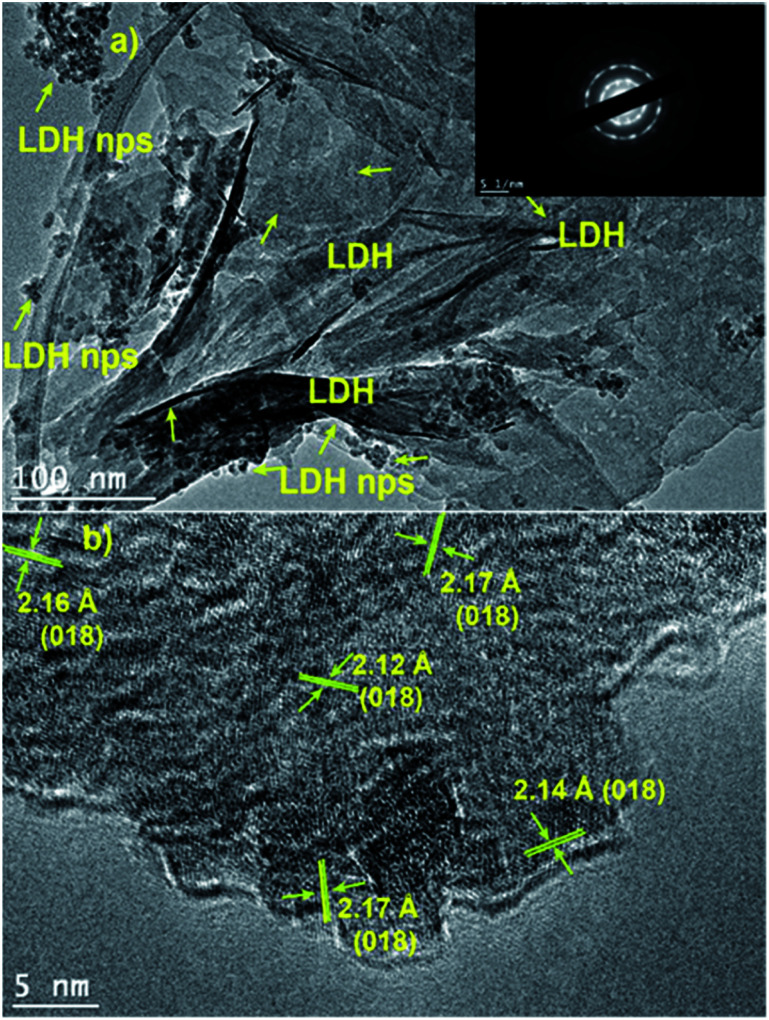
(a) TEM and selected area electron diffraction pattern of CoGaFe-LDH (b) HRTEM images of CoGaFe-LDH.

Fig. 6 shows the TEM and HRTEM images of CoGaFe-LDH. In Fig. 6a we see that the morphology for CoGaFe-LDH consists of nanosheets and nanoparticles. Additionally, we can see that the selected area electron diffraction (SAED) pattern shows a series of concentric rings, which indicates that the section is polycrystalline (inset of Fig. 6a). In Fig. 6b we measured a distance of 2.145 ± 0.025 Å, which agrees with the (018) plane of the hydrotalcite (JCPDS: 41-1428). The micrographs of the other materials are presented in the Fig. S10–S13.† We could also observe that Zn-containing materials have a very small amount of rich in cobalt nanorods and nanoparticles of LDHs (Fig. S12 and S13†). These results are in agreement with those of XRD in that both of them show characteristic planes of the hydrotalcite (JCPDS: 41-1428). Due to the last observation, we attributed the absence of nanorods and nanoparticles mainly to the presence of Zn^2+^.

**Fig. 7:**
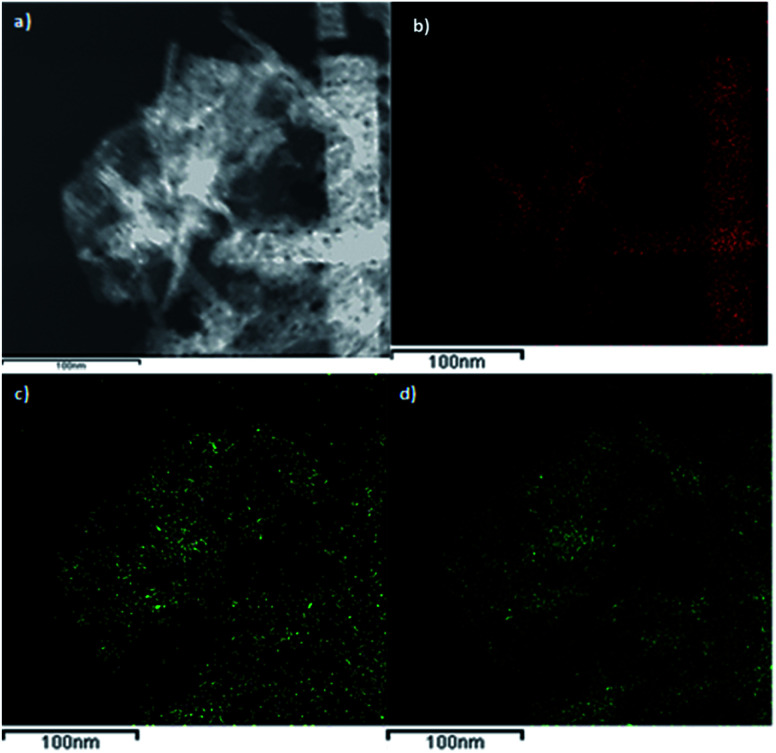
Chemical mapping of CoGaFe-LDH. (a) Dark-field micrograph. (b) Co, (c) Ga, and (d) Fe chemical mappings.

Fig. 7 shows a dark-field TEM micrograph of the CoGaFe-LDH and its chemical mapping. We observed that CoGaFe-LDH is composed of Fe, Co, and Ga confirming that the metal elements were adequately incorporated in this material. Furthermore, it is observed that the nanorod on the right side of Fig. 7a, is composed mainly of Co (as seen in Fig. 7b), as long as the Ga and Fe are not observed (Fig. 7c and d). We also confirmed the morphology showed in the TEM and HRTEM micrographs, as well as we observed in all the materials, nanoparticles, and agglomerates of nanoparticles rich in iron (Fig. S14–S22†).

Samples with higher cobalt loading promoted the formation of Co(OH)_2_ nanorods, as in the CoGaFe-LDH sample (Table S2†).

### Electrochemical performance

**Fig. 8:**
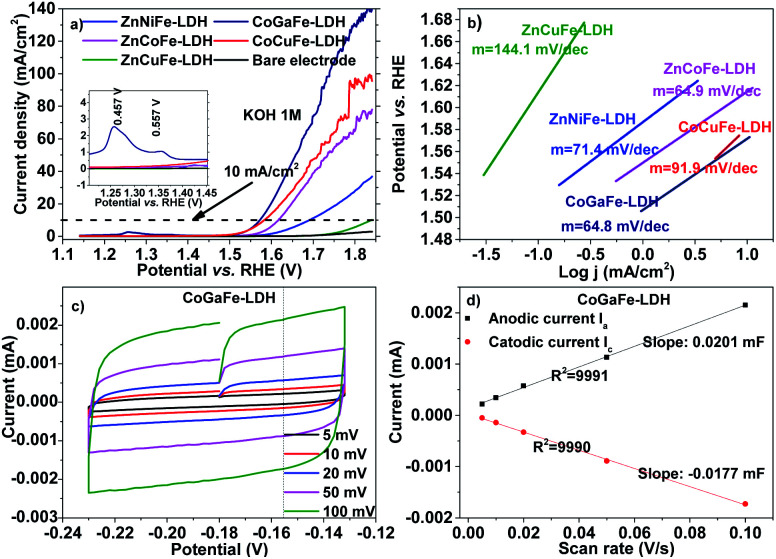
(a) LSV measurements for the synthesized LDHs. (b) Tafel slopes, (c) and (d) double-layer capacitance measurements to determine the electrochemically active surface area for the CoGaFe-LDH catalyst.

Fig. 8 presents the electrocatalytic measurements that were carried out through LSV and CV. In Fig. 8a, the linear sweep voltammograms of the materials are shown. The overpotential “*η*” was calculated using the eqn (4):

4 *η* = *E*_RHE_ − 1.23 V

where *E*_RHE_ is the potential in the scale of the reversible hydrogen electrode. We observed that the catalyst with the lower overpotential is the CoGaFe-LDH, reaching 369.3 mV at a current density of 10 mA cm^−2^. In the inset of Fig. 8a we can observe two oxidation peaks located at 457 and 557 mV. The first one appears in the oxidation potential of Fe^0^ to Fe^2+^ process and the second one appears at the oxidation potential of Ga^0^ to Ga^3+^. These oxidations would cause a positive electronic density in the material surface, which would favor the adsorption of water molecules or the charge transfer from the water molecules to the material, thus improving activity toward the WOR.

Fig. 8b shows the Tafel slopes derived from LSV measurements in Fig. 8a according to the Tafel equation (eqn (5)), by plotting the overpotential (or the potential) against the logarithm of the current density.

5 *η* = *b* log(*j*)

where *b* is the Tafel slope. Through these measurements, we observed that the catalysts containing Zn^2+^ and Cu^2+^ reaching slopes higher than the other materials, indicating low kinetics, while the materials with Co^2+^ improved their kinetics reaching values around 64.8 mV dec^−1^.^47^ A comparison of the electrocatalytic properties of the as-synthesized LDHs is shown in Table S7.† The values of overpotential and Tafel slope of CoGaFe-LDH is comparable to the most of those hydroxides, which were tested on several of electrodes such as GCE, carbon cloth (CC), carbon fibers (CF), as well as nickel foam (NF).^8^

The capacitance of the electrical double layer was determined from CV recorded at the non-faradaic potential as the average of the absolute values of the slopes as shown in Fig. 8c and d. The capacitive current is related to the scan rate through eqn (6):

6 *I*_c_ = *νC*_dl_

where “*I*_c_” is the current to charge the double layer, *ν* is the scan rate, and “*C*_dl_” is the double-layer capacitance.^13^

Cyclic voltammograms, as well as the current *vs.* scan rate graphs for the other electrocatalysts are shown in Fig. S23 and S24.† Owing to there is a lack of an accurate and feasible method to measure the specific capacitance of the material, and knowing that the electrochemically active surface area (ECSA) is related to the specific capacitance by the eqn (7) and (8):

7 SRF = *C*_dl_/*C*_s_

8 ECSA = SRF × *A*_g_

where “SRF” is the surface roughness factor, “*C*_s_” is the specific capacitance, and “*A*_g_” is the geometric area of the electrode. We can qualitatively determine which material has the highest ECSA.^47^ Besides, there are reports related to these materials, where the value of 0.040 mF cm^−2^ to specific capacitances is used for KOH and NaOH electrolytes.^13^ Thus, we determined an ECSA value for the synthesized LDHs, using the reported specific capacitance and the capacitance measured by CV. These results show that materials with Zn^2+^, Cu^2+^, or Ni^2+^ had lower ECSA values than materials with Co^2+^ or Ga^3+^ which had higher values.

**Fig. 9:**
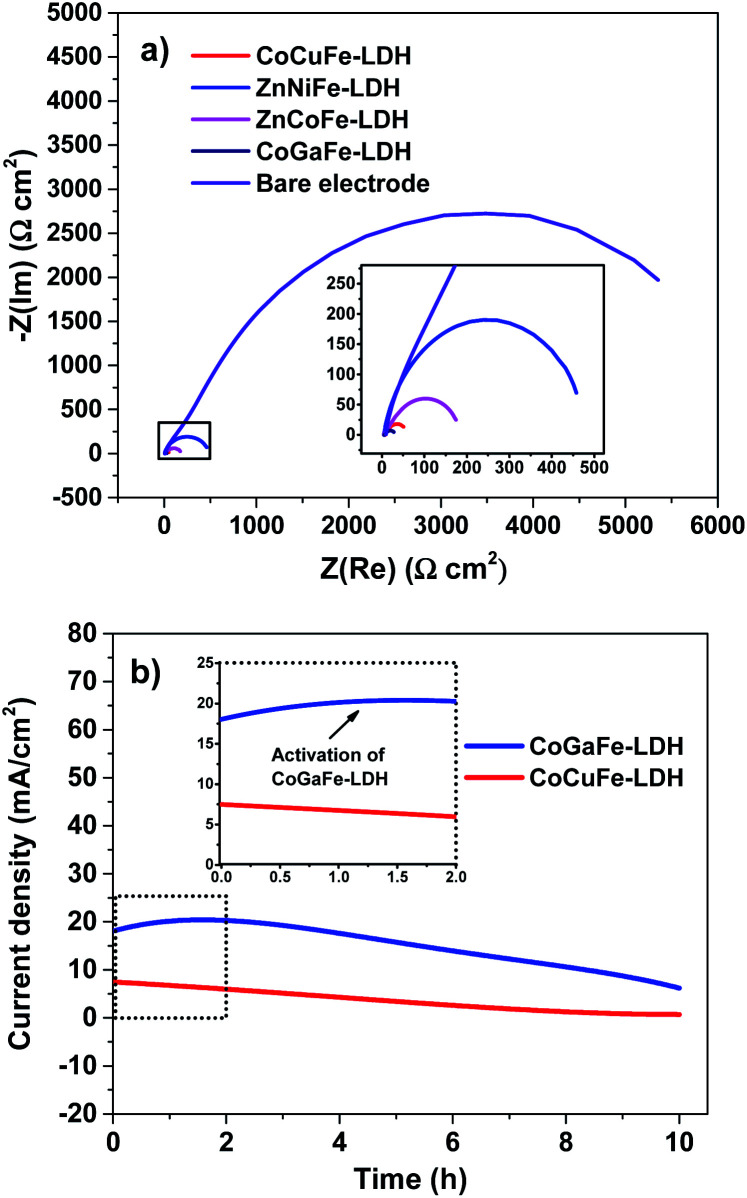
(a) Nyquist plots for synthesized LDHs. (b) Chronoamperometric measurements of CoGaFe-LDH and CoCuFe-LDH.

We also measured the electrode kinetics through the charge transfer resistance (*R*_ct_), which was obtained through Nyquist plots of the LDHs (Fig. 9a). From this plot, we observe the typical semicircles that are correlated with the *R*_ct_ as follow; the smaller the semicircle the more active electrocatalytically the catalyst is. We noticed that the resistance due to the charge transfer in the materials follows the order: ZnNiFe-LDH > ZnCoFe-LDH > CoCuFe-LDH > CoGaFe-LDH. Hence, we concluded that Zn^2+^, Cu^2+^, or Ni^2+^ had higher *R*_ct_ values than materials with Co^2+^ or Ga^3+^ which had lower *R*_ct_ values. The lowest *R*_ct_ was achieved by the CoGaFe-LDH reaching values as low as 30.1 Ω cm^2^. The decrease in the *R*_ct_ in these materials could be due to the electronic effect of having a Ga^3+^ cation in octahedral coordination with OH^−^ anions as we will see below.^48^

Table 4 show all electrocatalytic parameters calculated for the synthesized trimetallic LDHs. The values of *R*_ct_ were calculated with the value measured by the Nyquist plot and then dividing by the ECSA. We noted that the materials with Zn^2+^ are the fewer actives, which suggests that Zn^2+^ could act as an inhibitor for this reaction. This could be due to Zn^2+^ has a closed electronic configuration, therefore it is more difficult to transfer the electronic charge, and the crystal field that this cation exerts in this coordination and oxidation state. On the other hand, the CoGaFe-LDH is the more active material which suggested that Ga^3+^ is the best promotor for WOR. In this case, despite the Ga^3+^ has the same electronic configuration than Zn^2+^, the Ga^3+^ has one positive charge more than the Zn^2+^. Also, the Ga^3+^ has an ionic radius equal to 76 pm when it has a coordination number of 6 whereas the Zn^2+^ in these conditions has an ionic radius to 88 pm leading to a different crystal field strength. We attributed this improvement in the electrocatalytic activity to the electronic properties of the Ga^3+^.

**Table 4:** Electrocatalytic parameters of the LDHs synthesized

Material	*η* at 10 mA cm^−2^ (mV)	Tafel slope (mV dec^−1^)	*C*_dl_ (μF cm^−2^)	ECSA (cm^2^)	*R*_ct_ (Ω cm^2^)
CoCuFe-LDH	415.5	91.9	38.8	0.0686	59.6
ZnNiFe-LDH	488.0	71.4	32.2	0.0570	482.0
ZnCoFe-LDH	413.2	64.9	34.6	0.0612	235.2
ZnCuFe-LDH	636.9	144.1	32.6	0.0577	—
CoGaFe-LDH	369.3	64.8	37.8	0.0669	30.1

Fig. 9b shows the stability tests during 10 h for the two most active materials (CoGaFe-LDH and CoCuFe-LDH). The CoGaFe-LDH exhibited an increase of 14% in current density in the first two hours owing to an electrochemical activation (see inset of Fig. 9b). At the end of the experiment, the current density of CoGaFe-LDH drops to 66% of its initial value. In contrast, the second most active material (CoCuFe-LDH) presented a gradual loss of current density, which lost 88% of its activity. The decrease in catalytic activity can be due to the detachment of the catalyst from the electrode as O_2_ evolves.^49,50^ Additional studies should be done to investigate the deactivation pathways of these materials.

## Conclusions

In this work, we studied the electrocatalytic influence of Co^2+^, Cu^2+^, Ni^2+^, Zn^2+^, and Ga^3+^ on the ternary LDHs for WOR. UV-vis spectroscopy analyses showed that these materials absorb in the visible spectrum having *E*_g_ values from 1.45, to 2.45 eV for all the materials. Therefore, they could be applied to photocatalysis and photo-electrocatalysis fields. Through TGA analyses we notice that the Zn^2+^ is playing a significant role as a thermal stabilizer while Co^2+^ has the opposite effect. XPS O_1s_ spectra of the materials showed that the amount of oxygen vacancies follows the order: ZnCoFe-LDH > CoCuFe-LDH > CoGaFe-LDH > ZnNiFe-LDH > ZnCuFe-LDH. Nevertheless, the electrocatalytic activity follows the order: CoGaFe-LDH > CoCuFe-LDH > ZnCoFe-LDH > ZnNiFe-LDH > ZnCuFe-LDH. However, despite the number of oxygen vacancies enhances the electrocatalytic activity toward the WOR, we suggested that this is not only the main factor for the enhancement of trimetallic LDHs for WOR. Also, the XPS and UV-vis studies reveal that CoGaFe-LDH material does not have mixed oxidation states for the same element, which excludes the possibility that the catalytic activity is due to the mixed oxidation state. Although CoGaFe-LDH exhibited lower specific surface area than that of ZnNiFe-LDH (Table 1), it showed better catalytic activity. Besides, the CoCuFe-LDH catalyst has the lowest specific surface area and is the second material more active among the synthesized samples.

The above evidence the role of Ga^3+^ cation in enhancing the catalytic activity of the materials to improve the performance of these catalysts. The material that contains the highest amount of Fe on the surface (CoCuFe-LDH), is the second most active material, in the case of the material with the highest activity (CoGaFe-LDH), it is concluded that Ga^3+^ plays a role of promoter in this material. The Tafel plot analyses revealed that materials with Zn^2+^ and Cu^2+^ in their composition produce low kinetics whilst materials with Co^2+^ or Ga^3+^ in their composition yields better performance toward the WOR. The *R*_ct_ and ECSA values obtained from EIS and CV, respectively, suggest that Zn^2+^, Ni^2+^, and Cu^2+^ decrease the electrocatalytic activity whereas the Co^2+^ or Ga^3+^ cations have the opposite effect. The presence of multi-valences and oxygen vacancies are not the main factors contributing to improve the reaction kinetics. However, the similarities in % vacancies and surface areas between the most active material (CoGaFe-LDH) and others of regular activity, indicate that there is a fourth factor that is promoting the dropping of energy barriers by decreasing the resistance to charge transfer. We believe that theoretical calculations could help us to understand this phenomenon. This new strategy to design trimetallic catalysts could be tested in other reactions.

## Author contributions

David Yong: synthesis, characterization, writing, discussion, edition, data analysis and treatment, original draft, review. Ricardo Valdez: characterization, development of ideas, reviewing, edition. Miguel A. Armenta: development of ideas, reviewing, edition. Noé Arjona: development of ideas, reviewing and edition. Georgina Pina-Luis: development of ideas, reviewing and edition. Amelia Olivas: project management and administration, founding acquisition, supervision, reviewing and edition.

## Conflicts of interest

There are no conflicts to declare.

## Supplementary Material

RA-012-D2RA01980A-s001
