# Supplementary material for: Influence of Co2+, Cu2+, Ni2+, Zn2+, and Ga3+ on the iron-based trimetallic layered double hydroxides for water oxidation
Source: RSC Adv. 2022 Jun 8;12(26):16955–65. doi: 10.1039/d2ra01980a (PMC9174971; doi:10.1039/d2ra01980a)
Supplement: RA-012-D2RA01980A-s001 [file RA-012-D2RA01980A-s001.pdf]

Electronic Supplementary Material (ESI) for RSC Advances.
This journal is © The Royal Society of Chemistry 2022

Supporting Information

Influence of Co^{2+}, Cu^{2+}, Ni^{2+}, Zn^{2+}, and Ga^{3+} on the iron-based trimetallic layered double hydroxides for water oxidation

Jesus David Yong,[a] Ricardo Valdez,[b] Miguel Ángel Armenta[c], Noé Arjona[d], Georgina Pina-Luis[b] and Amelia Olivas[a*]*

a. Centro de Nanociencias y Nanotecnología–UNAM, km. 107 Carr. Tijuana-Ensenada, C.P. 22860, Ensenada, B.C. Mexico.

b. Tecnológico Nacional de México/Instituto Tecnológico de Tijuana, Centro de Graduados e Investigación en Química, Blvd. Alberto Limón Padilla S/N, Mesa de Otay, C.P. 22500, Tijuana, B.C. Mexico.

c. Universidad Estatal de Sonora, Departamento de Ingeniería en Geociencias, Av. Niños Héroes, San Javier, C.P. 84160, Magdalena de Kino, Son. Mexico.

d. Centro de Investigación y Desarrollo Tecnológico en Electroquímica, Parque Tecnológico Querétaro, Sanfandila, C.P. 76703, Pedro Escobedo, Qro. Mexico.

Corresponding authors:

*E-mail: ricardo.valdez@tectijuana.edu.mx, aolivas@cnyn.unam.mx

Phone: +521 (669) 1878982, (646) 175 06 50, Ext. 370.

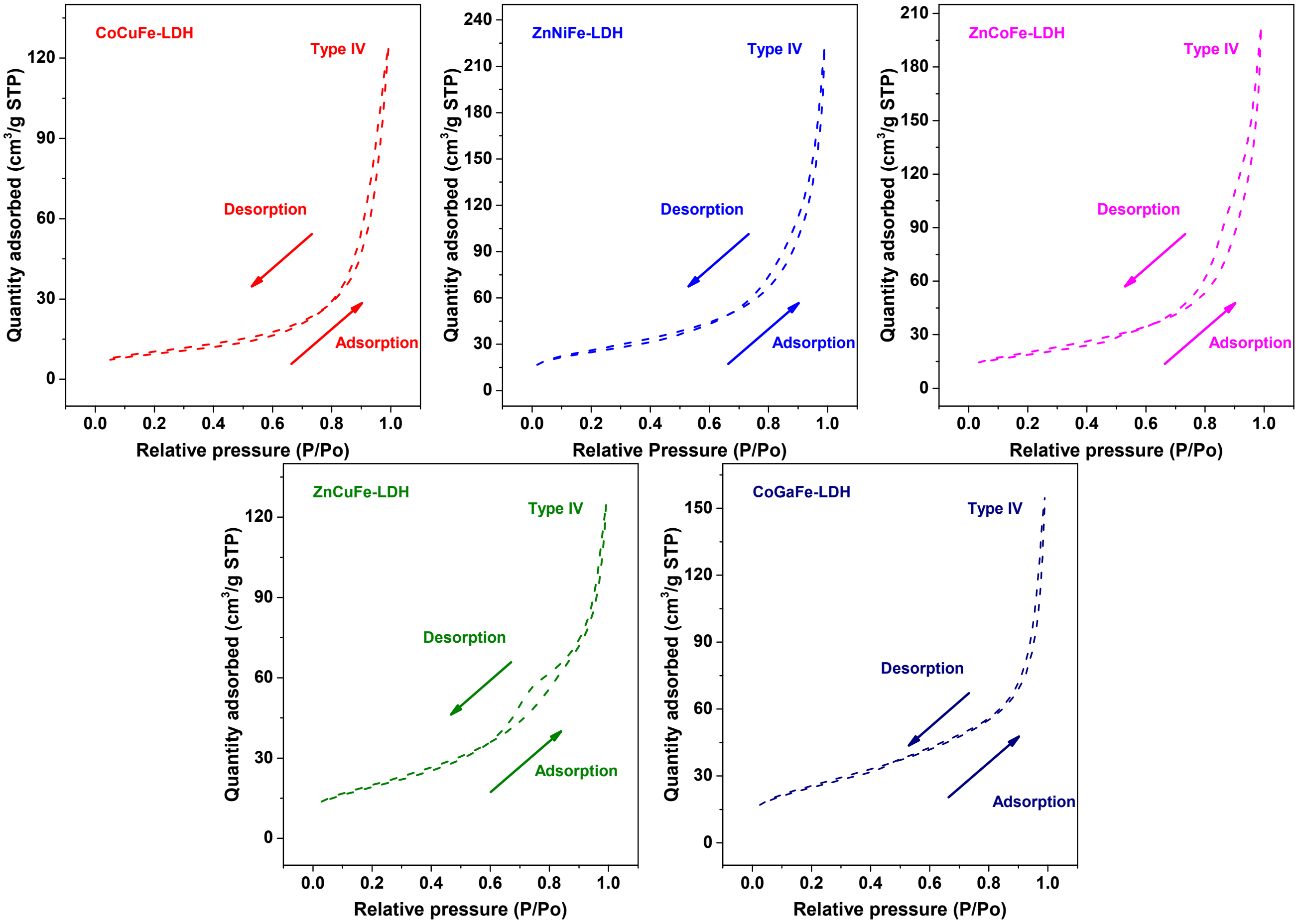

Fig. S1 N_2 Adsorption/desorption isotherms of the materials synthesized.

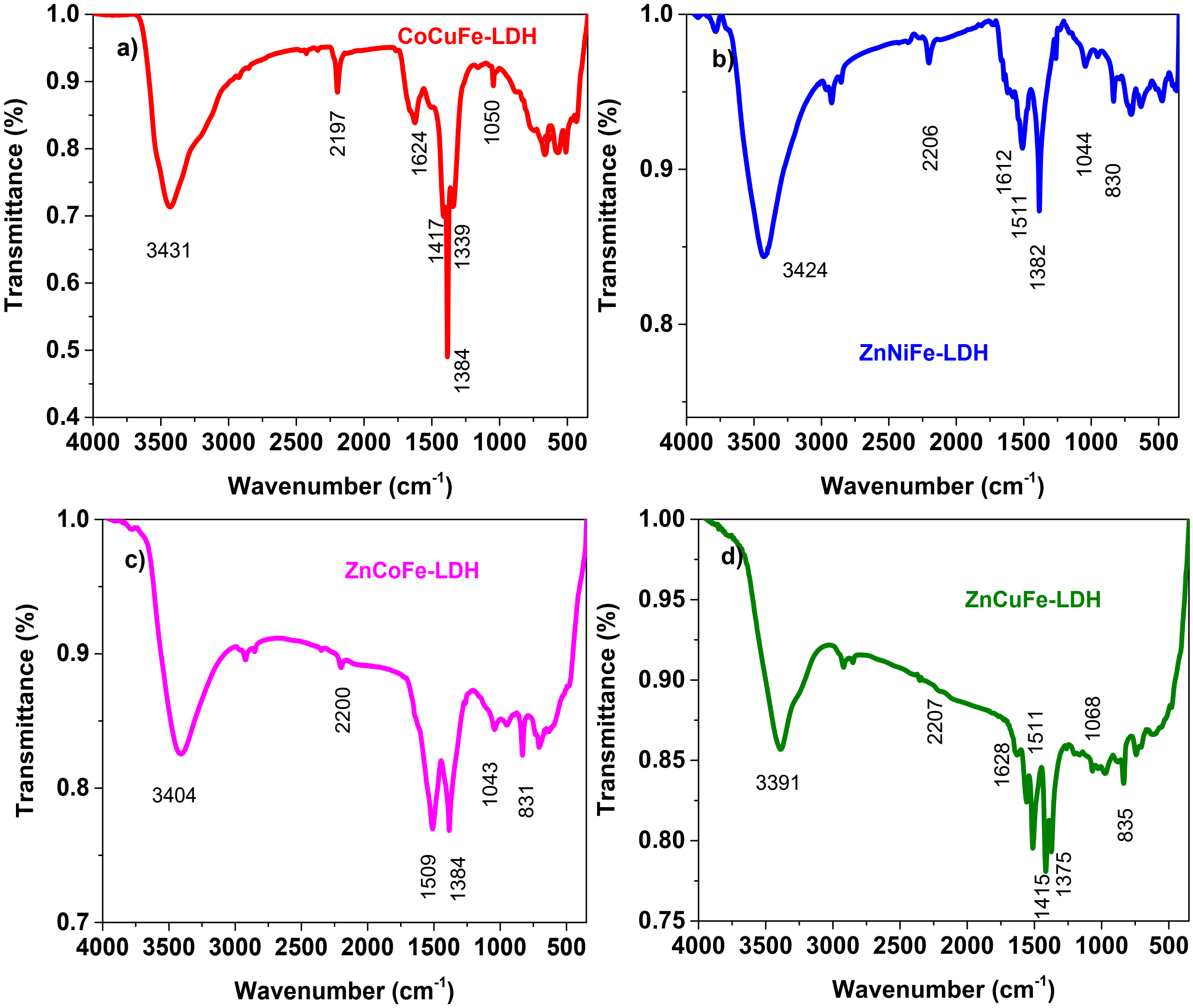

Fig. S2 FTIR spectra of a) CoCuFe-LDH, b) ZnNiFe-LDH, c) ZnCoFe-LDH, and d) ZnCuFe-LDH.

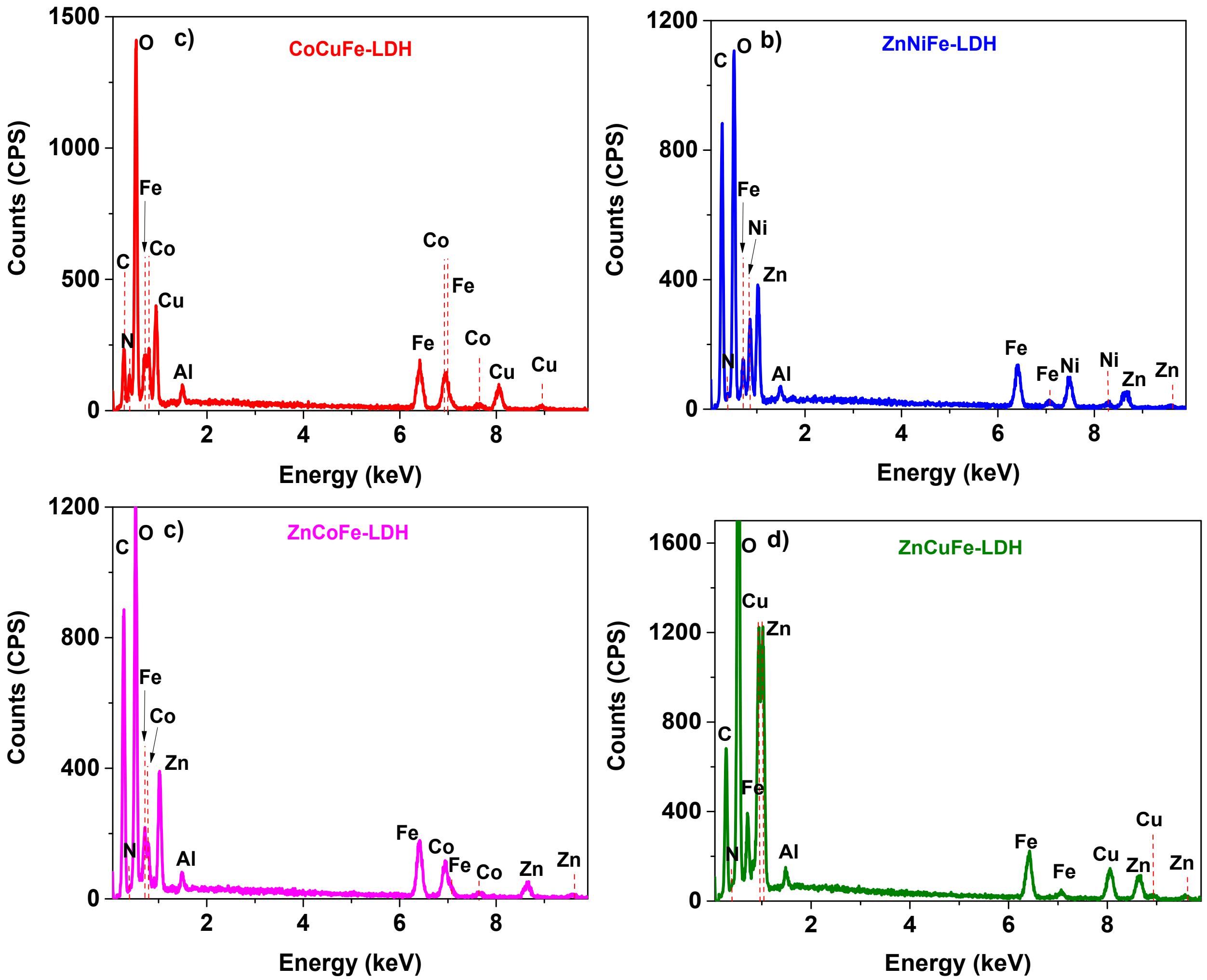

Fig. S3 EDS spectra of a) CoCuFe-LDH, b) ZnNiFe-LDH, c) ZnCoFe-LDH, and d) ZnCuFe-LDH.

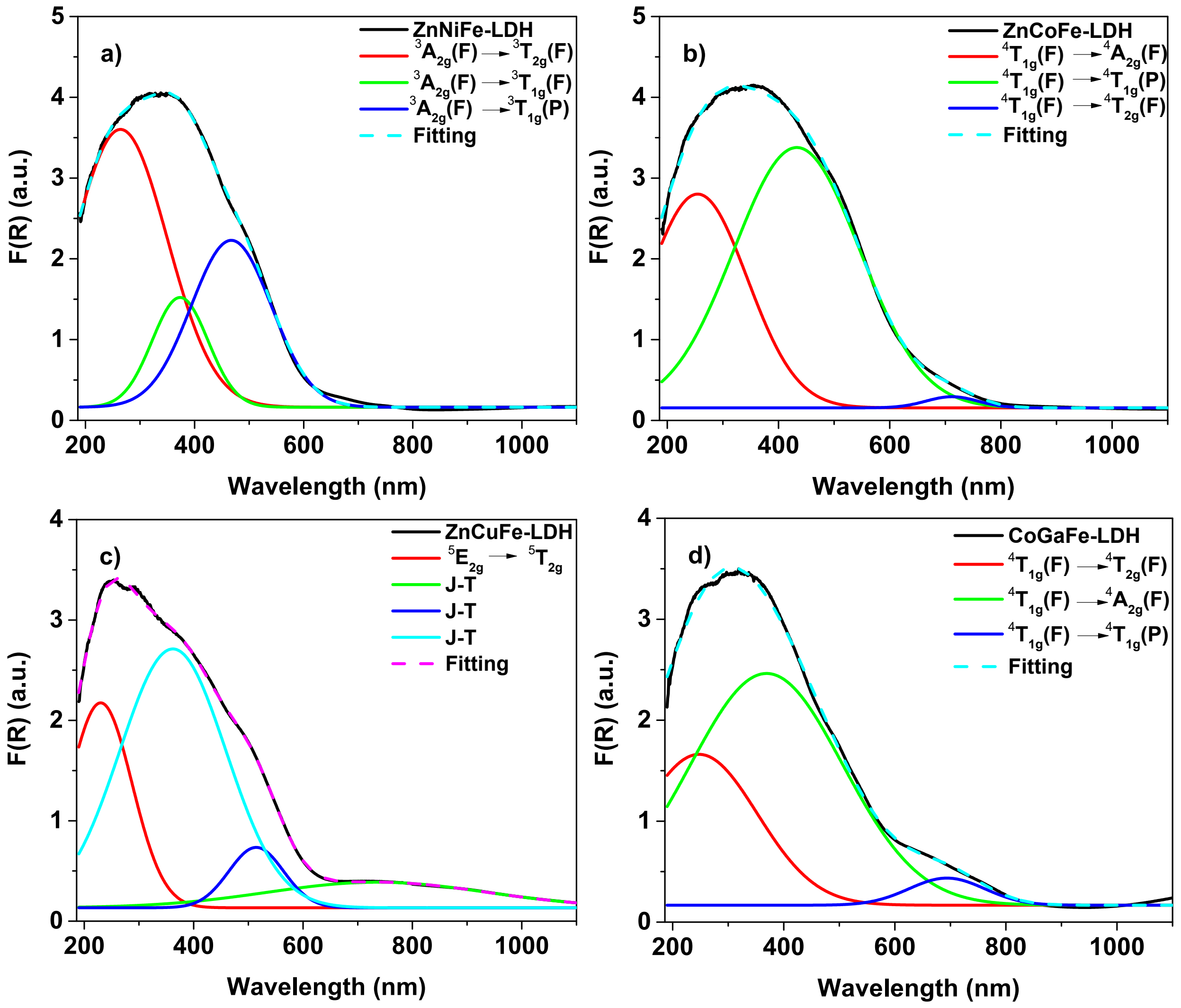

Fig. S4 UV-vis spectra of a) CoCuFe-LDH, b) ZnNiFe-LDH, c) ZnCoFe-LDH, and d) ZnCuFe-LDH.

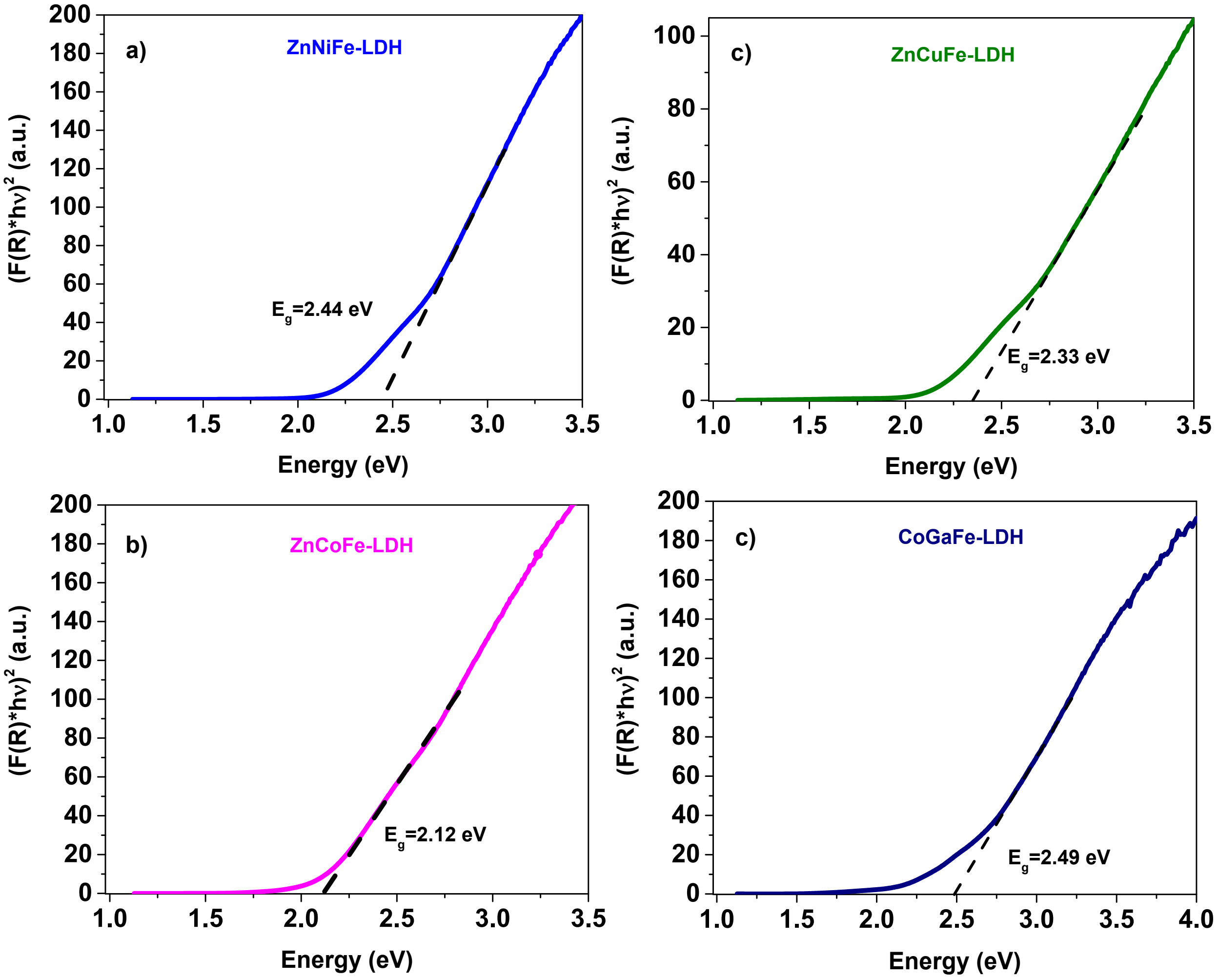

Fig. S5 Tauc plots of a) CoCuFe-LDH, b) ZnNiFe-LDH, c) ZnCoFe-LDH, and d) CoGaFe-LDH.

X–RAY PHOTOELECTRON SPECTROSCOPY

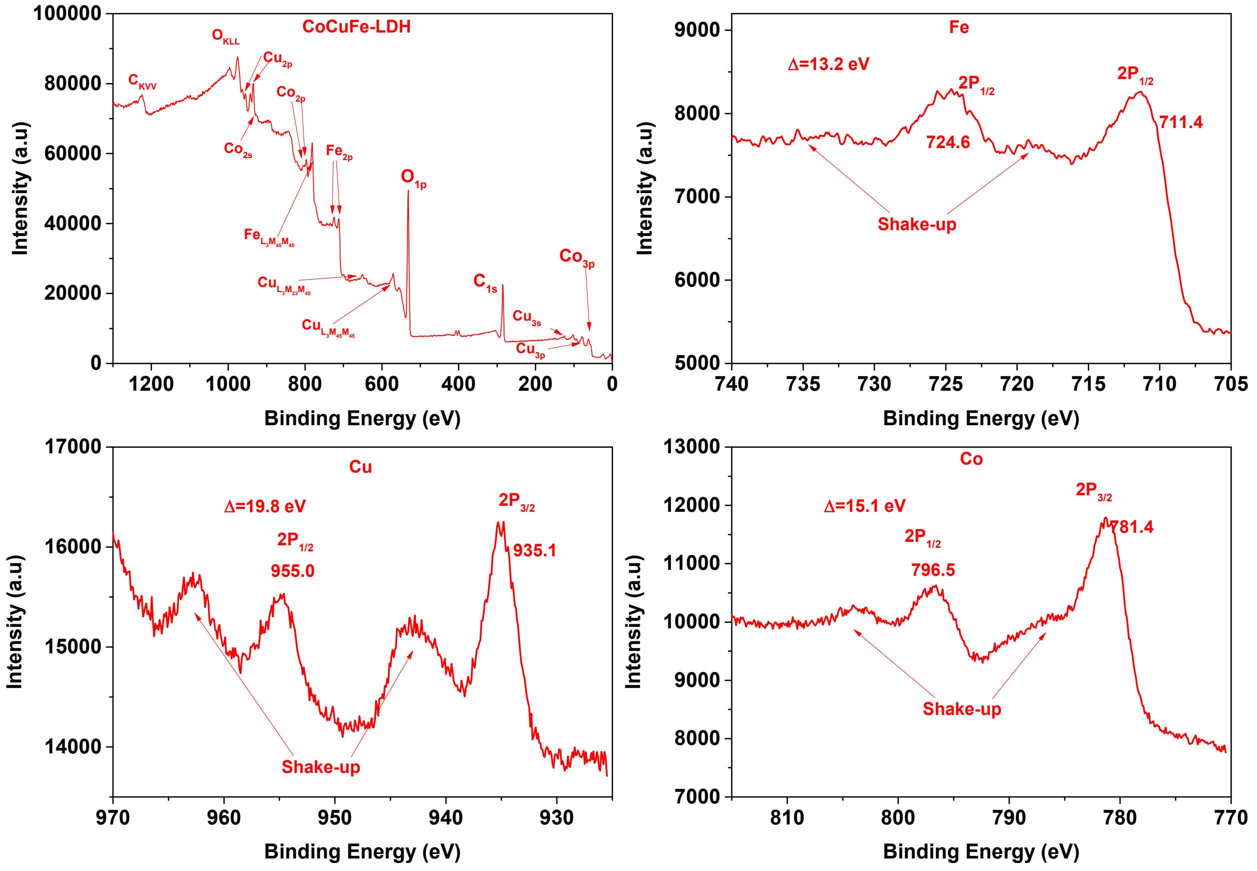

Fig. S6 XPS survey spectrum of a) CoCuFe-LDH. High-resolution spectra of b) Fe 2p, c) Cu 2p, and d) Co 2p.

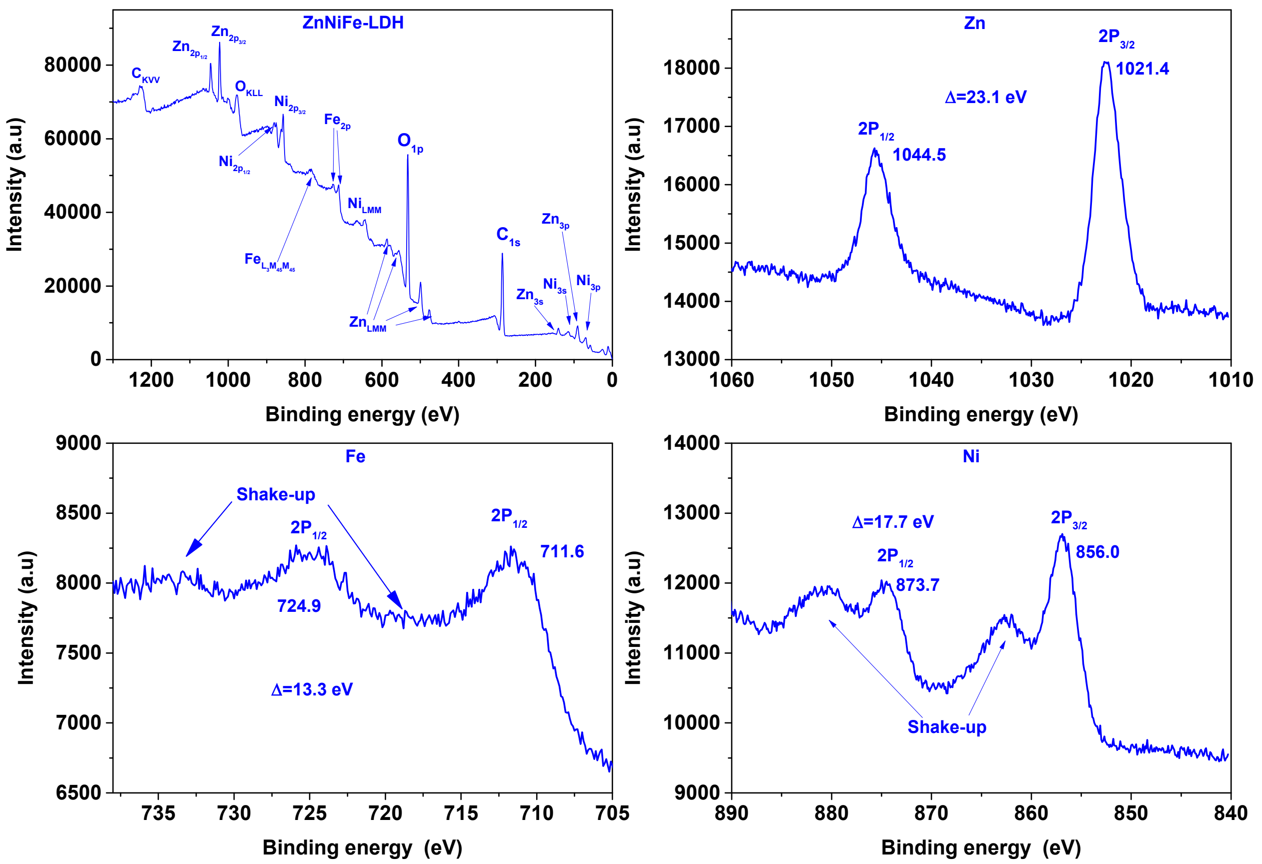

Fig. S7 XPS survey spectrum of a) ZnNiFe-LDH. High-resolution spectra of b) Zn 2p, c) Ni 2p, and d) Fe 2p.

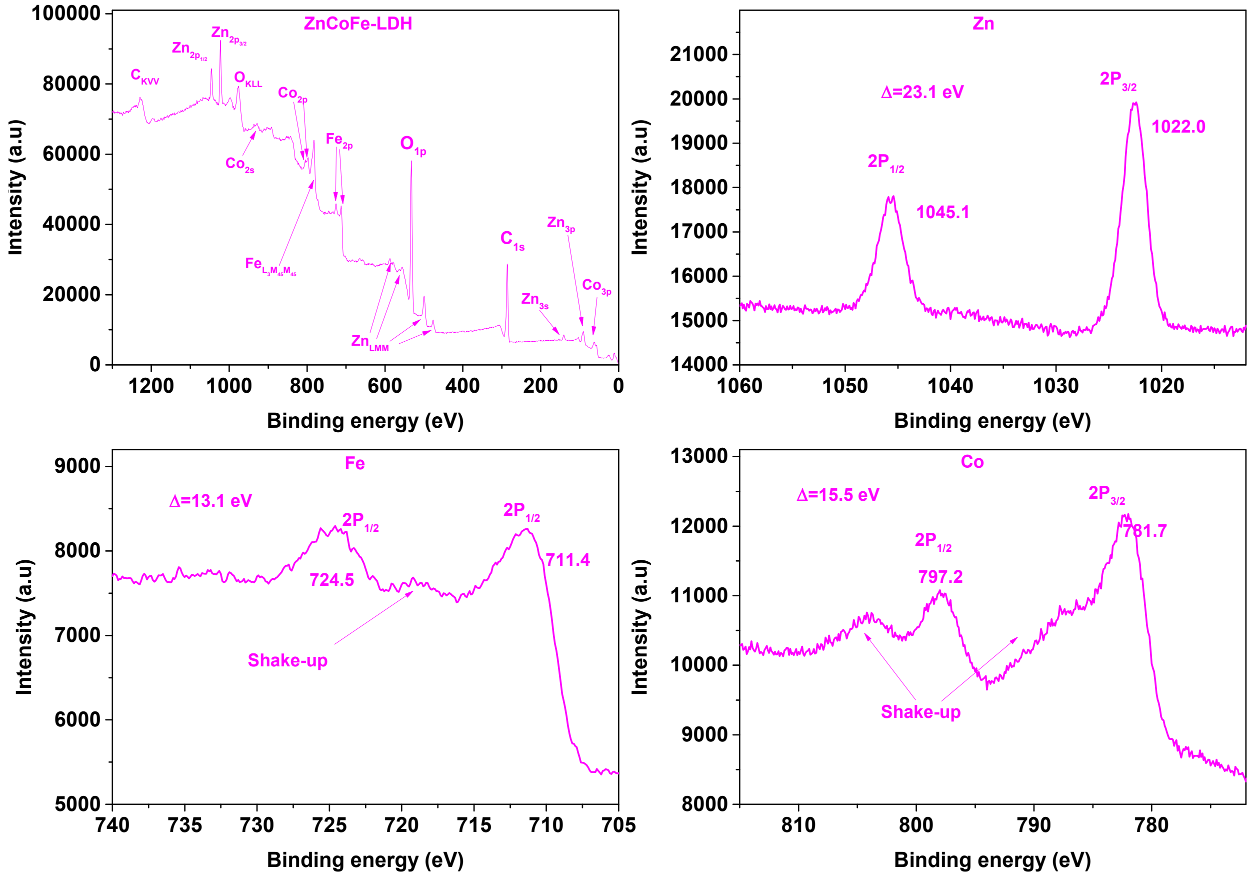

Fig. S8 XPS survey spectrum of a) ZnCoFe-LDH. High-resolution spectra of b) Zn 2p, c) Fe 2p, and d) Co 2p.

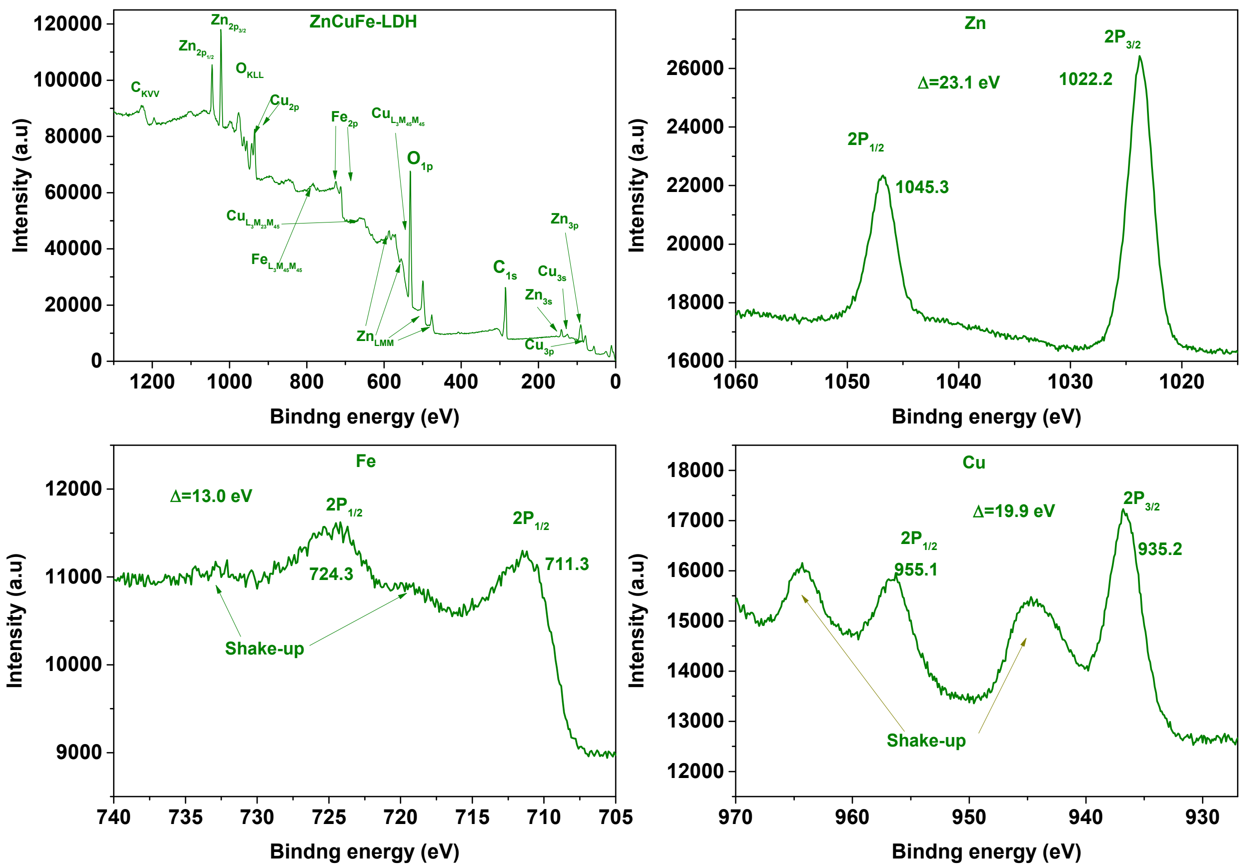

Fig. S9 XPS survey spectrum of a) ZnCuFe-LDH. High-resolution spectra of b) Zn 2p, c) Fe 2p, and d) Cu 2p.

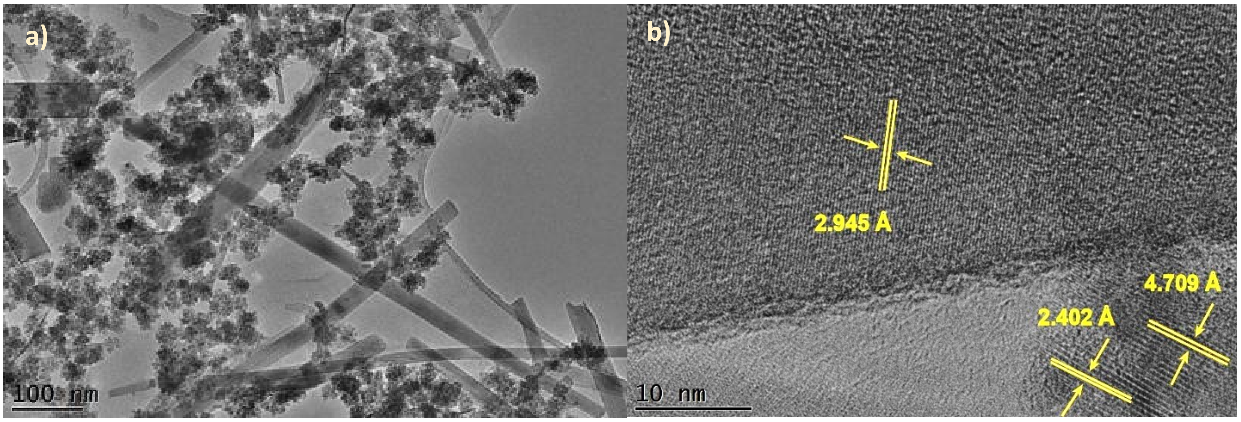

Fig. S10 Micrographs of TEM and HRTEM for CoCuFe-LDH. a) TEM micrograph and b) HRTEM micrograph for CoCuFe-LDH.

Fig. S10a shows that the morphology of CoCuFe-LDH consists of nanoparticles and nanorods. In the HR-micrograph (Fig. S9b) we determined the interplanar distances 2.945, 2.402 and 4.709 Å corresponding to the planes (001), (100), and (101), respectively, of $Co(OH)_2$ (JCPDS: 03-0913). Furthermore, the measured distances of 2.402 Å are in agreement with the distance of the hydrotalcite planes (015) (JCPDS: 41-1428).

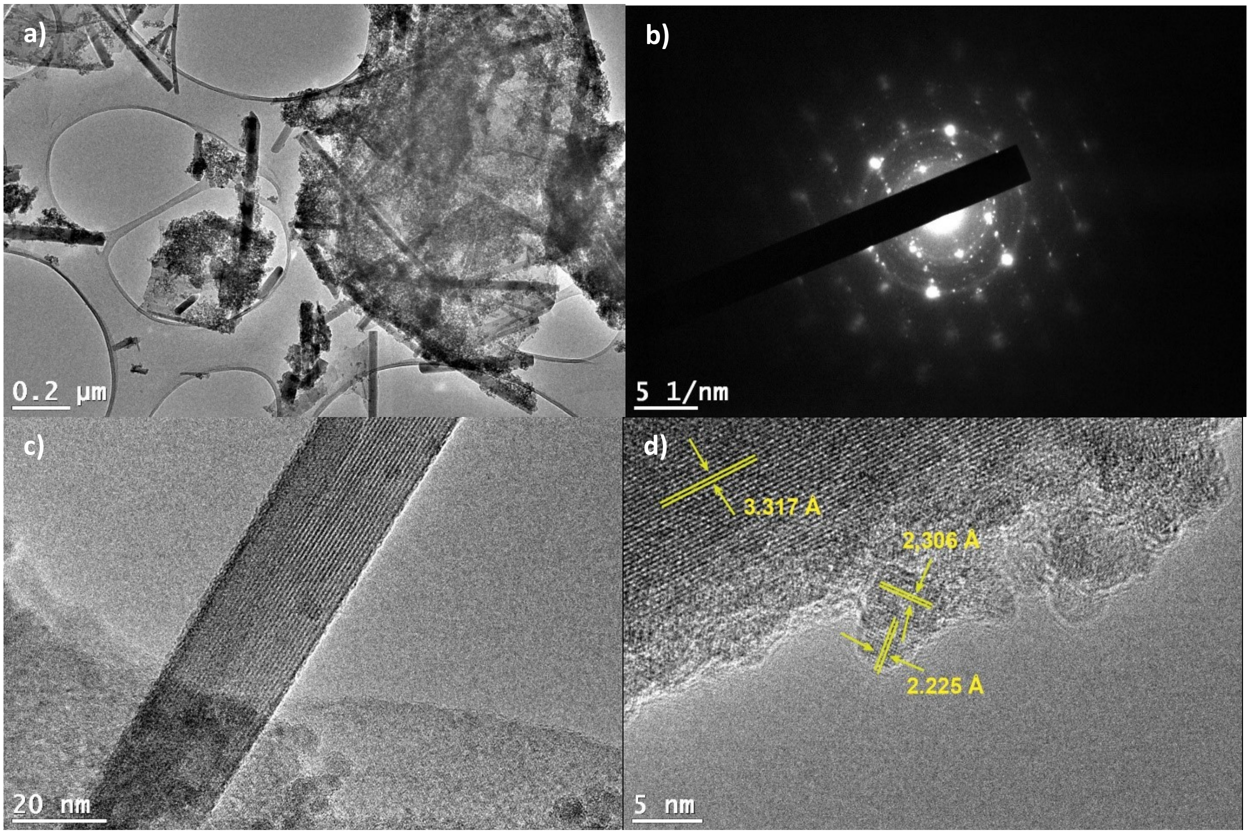

Fig. S11 TEM and HRTEM micrographs of the ZnNiFe-LDH. a) TEM micrograph, b) selected area electro diffraction pattern (SAED), c) HRTEM micrograph of a nanorod, and d) HRTEM micrograph.

Fig. S11a shows that ZnNiFeLDH is composed of nanocrystals and nanorods. In Fig. S11b we can observe the SAED pattern, which is the characteristic pattern of the hexagonal crystal system typical of hydrotalcite-like compounds. Fig. S11c shows the TEM micrograph of a nanorod, and Fig. S11d shows the typical interplanar distances of the hydrotalcite-like compounds.

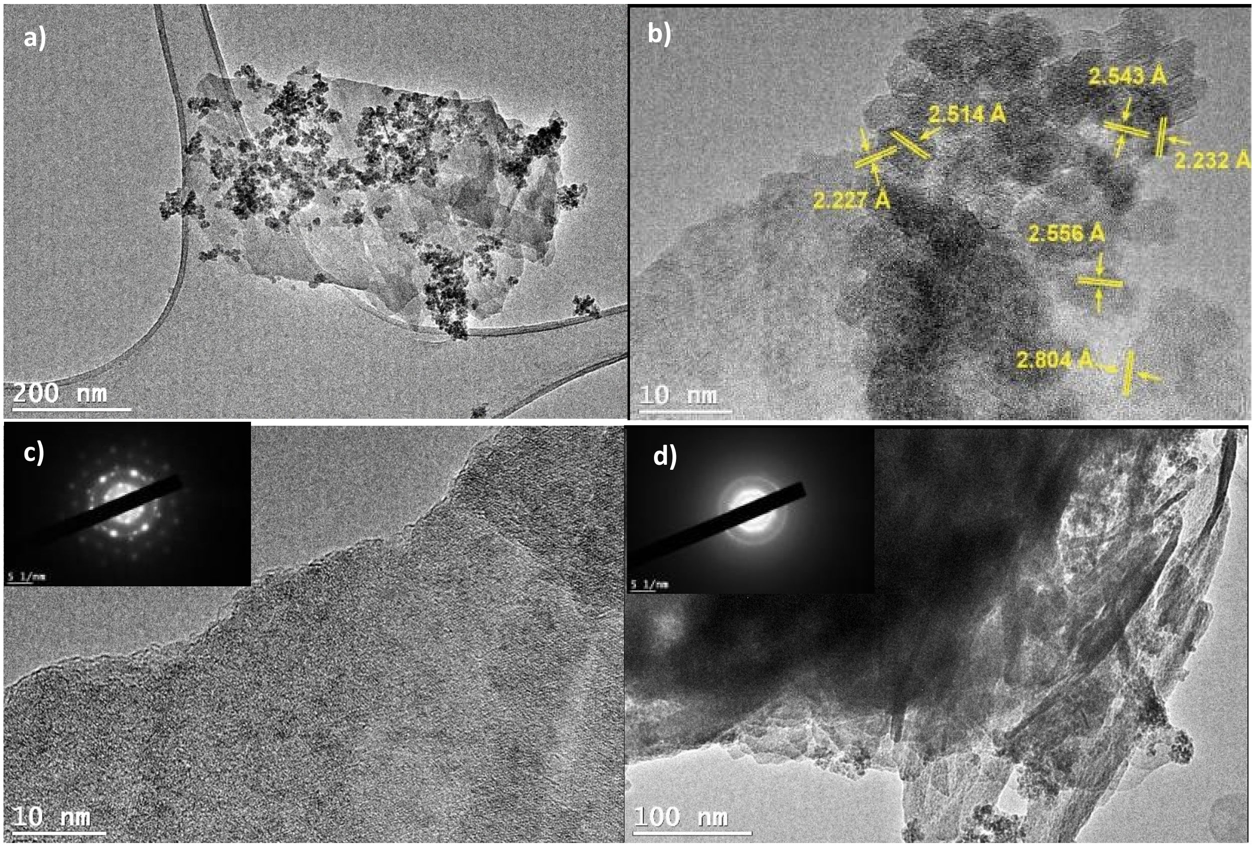

Fig. S12 TEM and HRTEM micrographs of the ZnCoFe-LDH. a) TEM micrograph, b) HRTEM micrograph. c) HRTEM micrograph (inset: SAED pattern), d) TEM micrograph (inset: SAED pattern), and d) HRTEM micrograph (inset: SAED pattern).

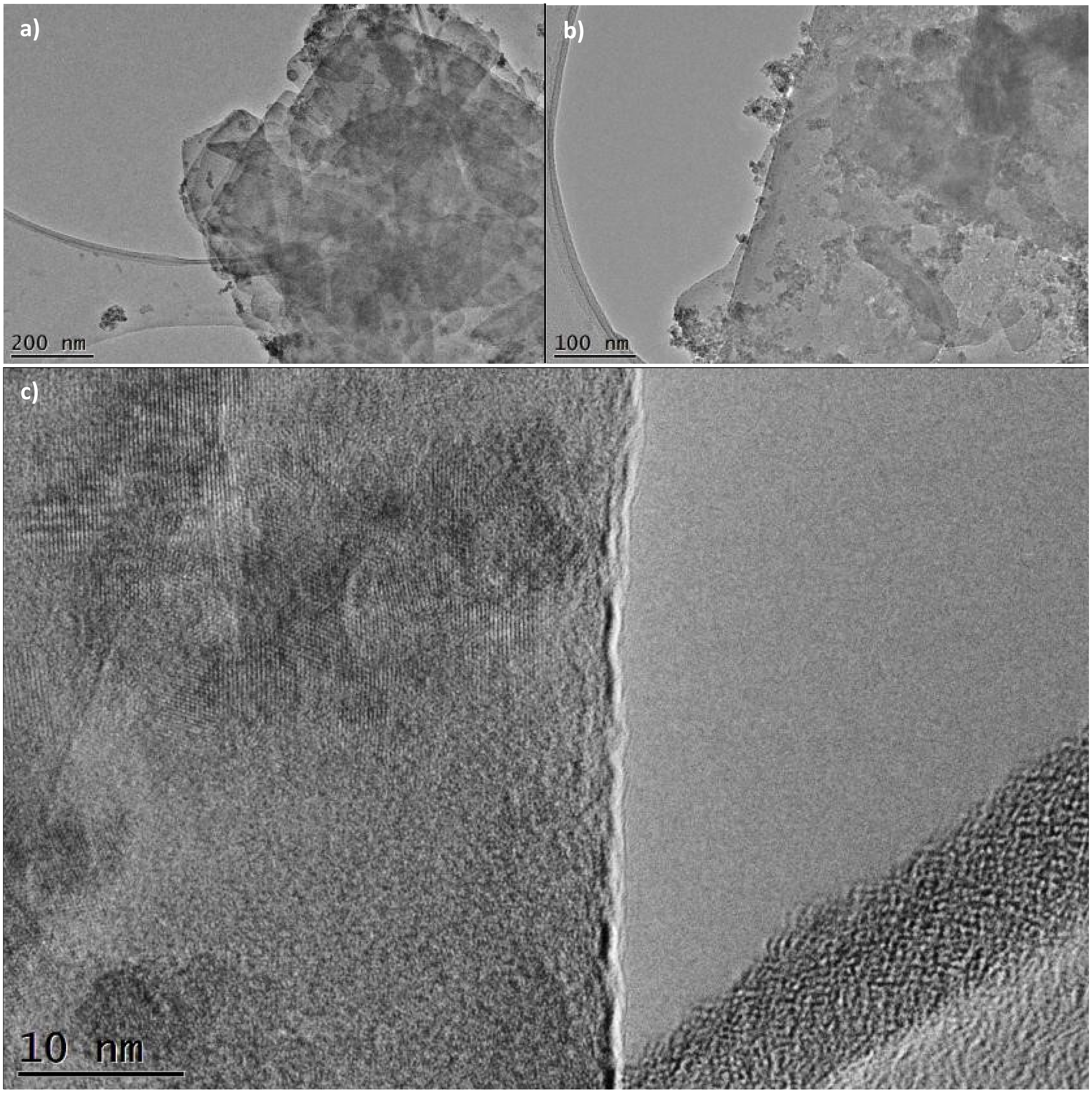

Fig. S13 TEM and HRTEM micrographs of the ZnCuFe-LDH. a) and b) TEM micrographs, and c) HRTEM micrograph.

In Fig. S13a and S13b we can note the lack of nanorods in the ZnCuFe-LDH material. Furthermore, we can also see that this sample has only a few amounts of nanoparticles. Fig. S13c shows the HRTEM micrograph.

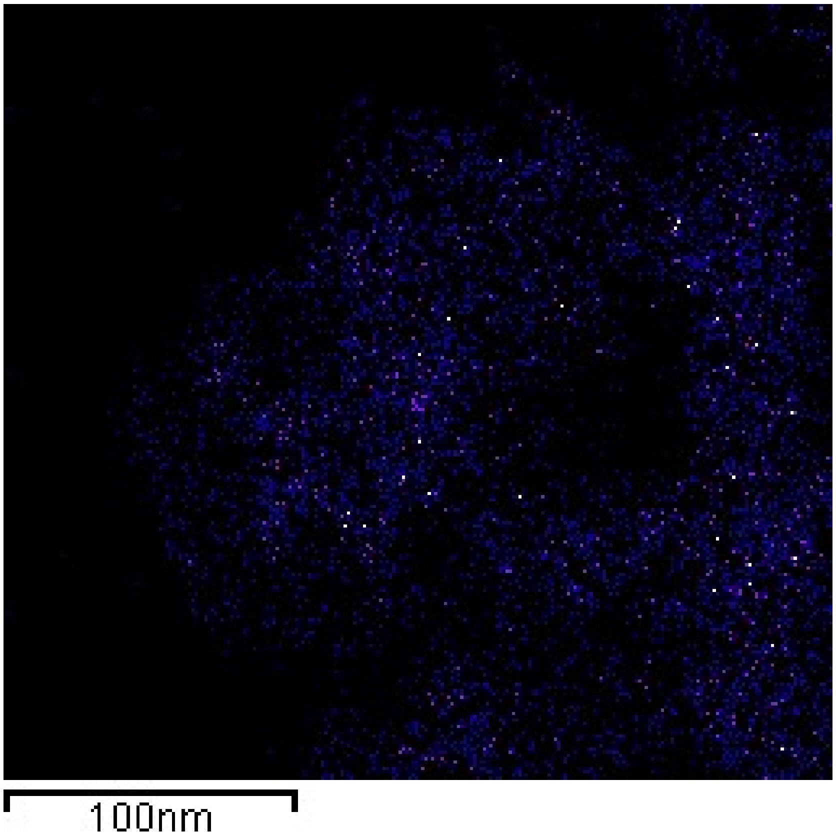

Fig. S14 Oxygen chemical mapping of CoGaFe-LDH.

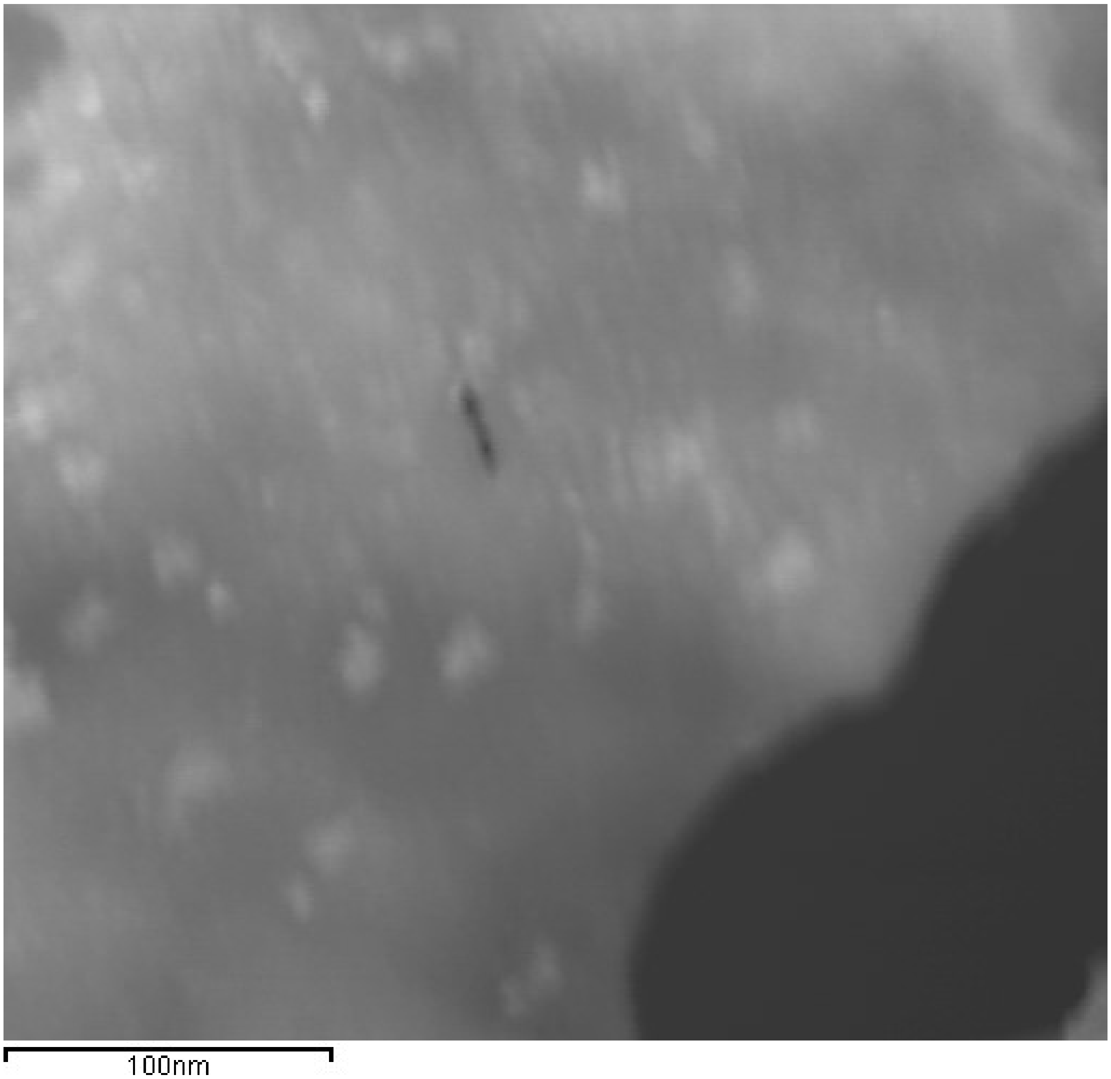

Fig. S15 Dark-field TEM micrograph of ZnNiFe-LDH.

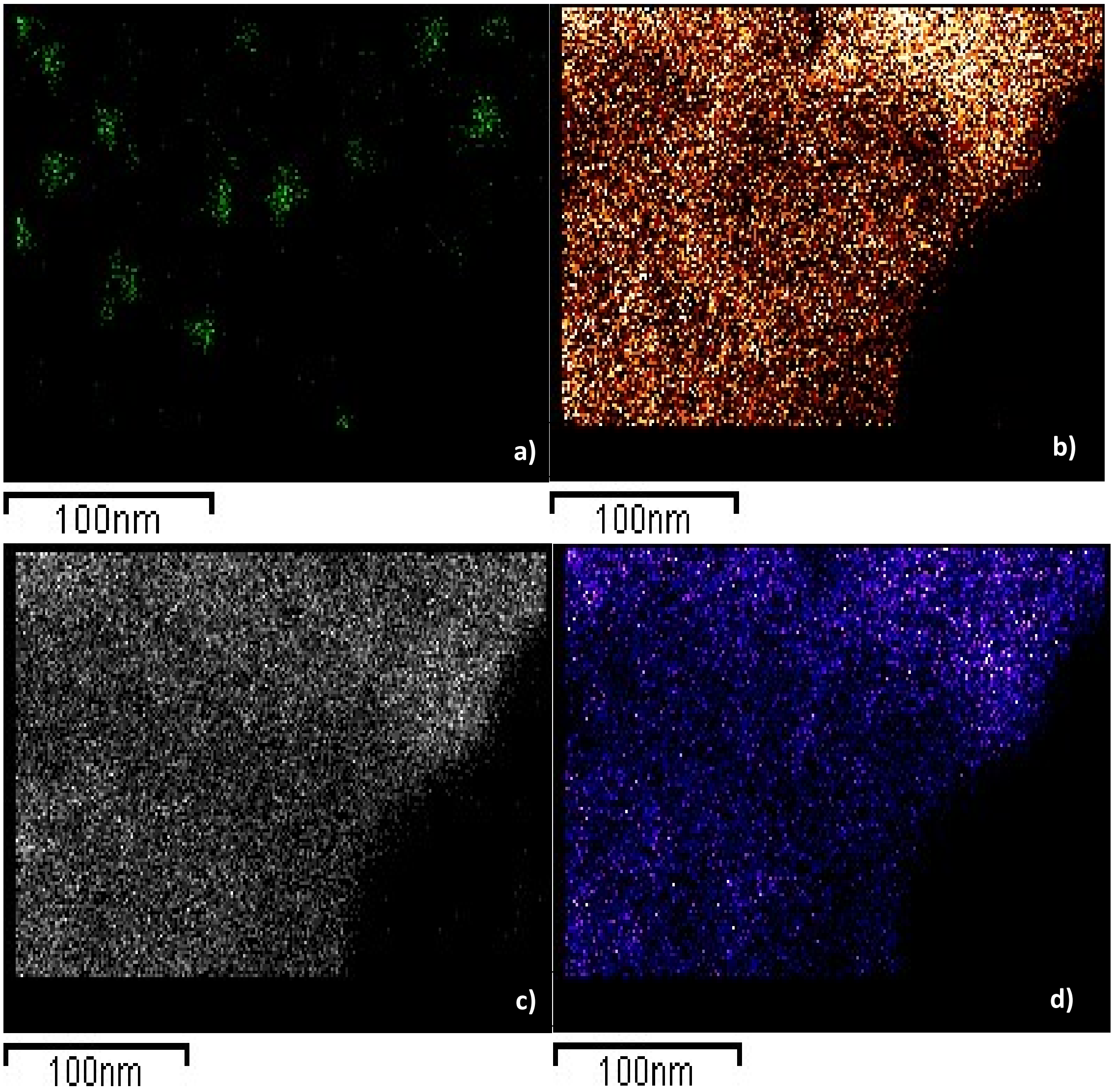

Fig. S16 Chemical mapping of ZnNiFe-LDH from the micrograph on Fig. S14. a) Fe, b) Zn, c) Ni and d) O.

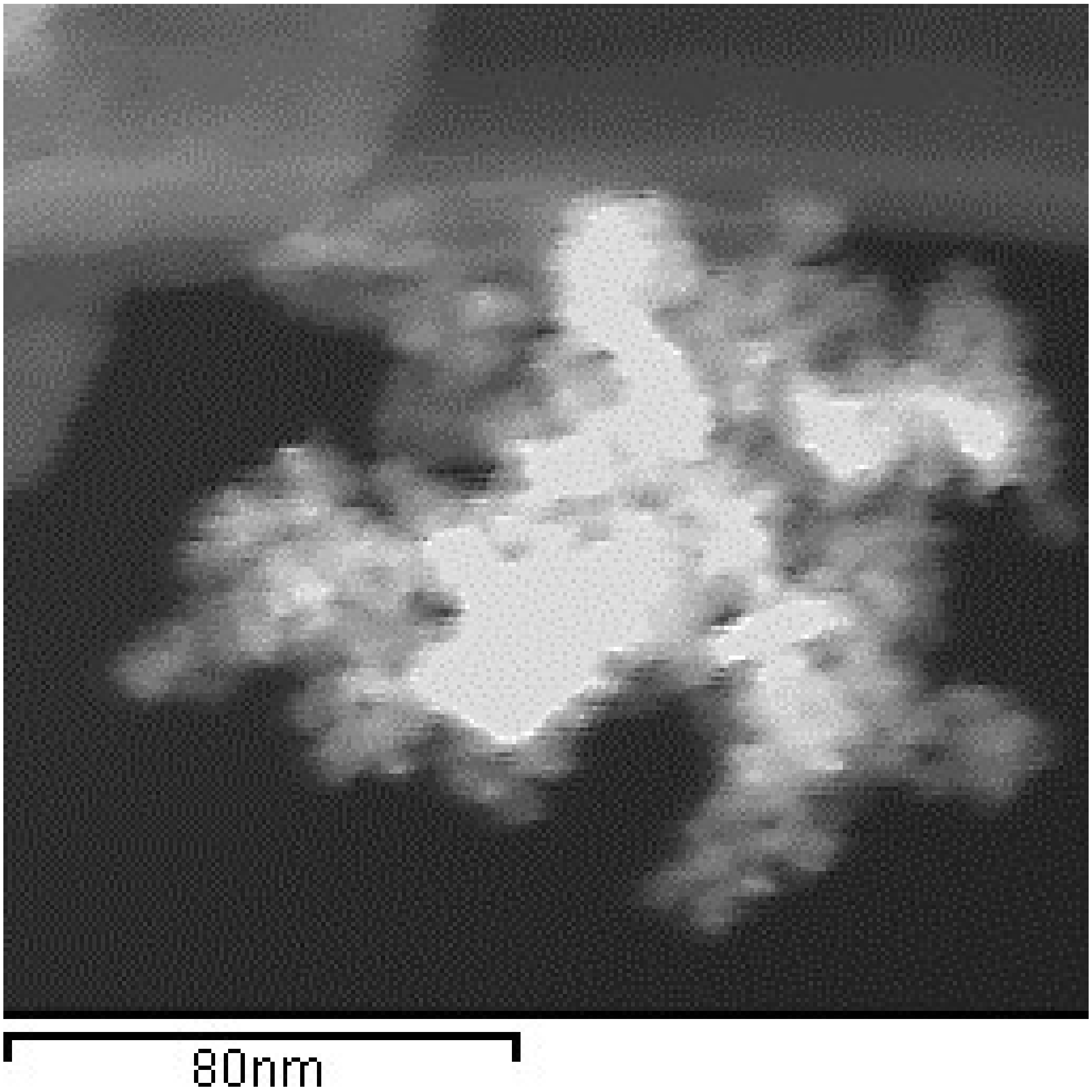

Fig. S17 Dark-field TEM micrograph of ZnCoFe-LDH.

Fig. S17 and S18 show the dark-field TEM micrograph of ZnCoFe-LDH and chemical mapping (Zn, Co, Fe, and O), respectively. We can see 2 kinds of morphologies, the first one in the center of the Fig. S17 (white color), while the second one in the upper and left side of the Fig. S17 (grey color). The white color zone shows that the material consists of agglomerated nanoparticles composed by Zn, Co, and Fe. However, we can note that the nanosheets in the upper and left side are composed mainly for Co and Zn. As we expected, oxygen is present in the whole sample.

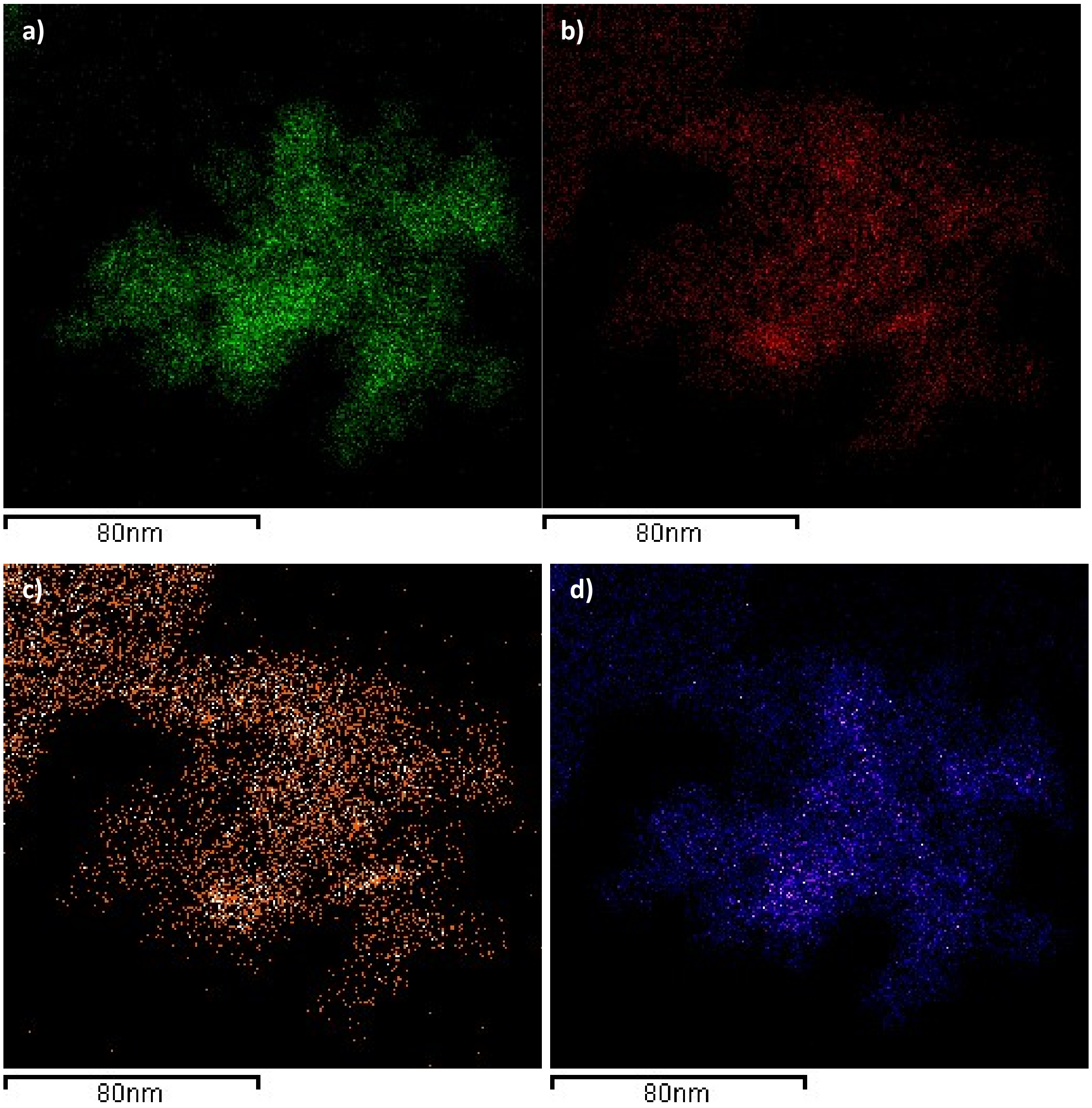

Fig. S18 Chemical mapping of ZnCoFe-LDH from the micrograph on Fig. S16. a) Fe, b) Co, c) Zn, and d) O.

It is important to say that in this case, the material does not show $Co(OH)_2$ nanorods, unlike CoCuFe-LDH and CoGaFe-LDH. This would support the hypothesis that $Co(OH)_2$ nanorods improve the performance toward WOR.

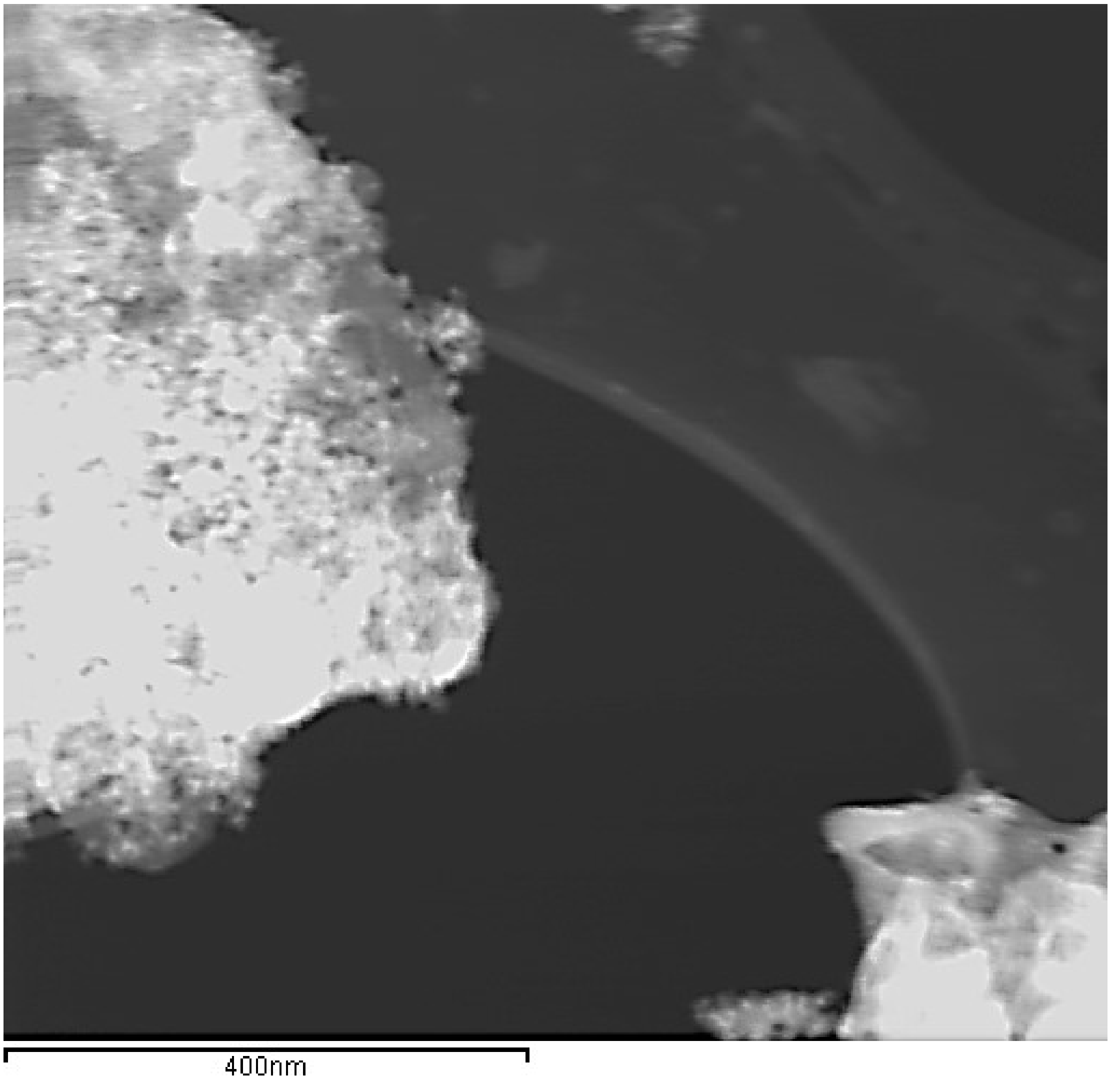

Fig. S19 Dark field TEM micrograph of ZnCuFe-LDH.

Fig. S19 and Fig. S20 show dark-field micrograph and chemical mapping (Zn, Cu, Fe, and O) of ZnCuFe-LDH, respectively. In Fig. S19 we note that there are two clusters of material. Chemical mapping in Fig. S20 shows that the upper cluster is composed of a large amount of Fe than the lower one. Both clusters are composed of Zn, Cu, and Fe. As expected, oxygen is into the whole sample.

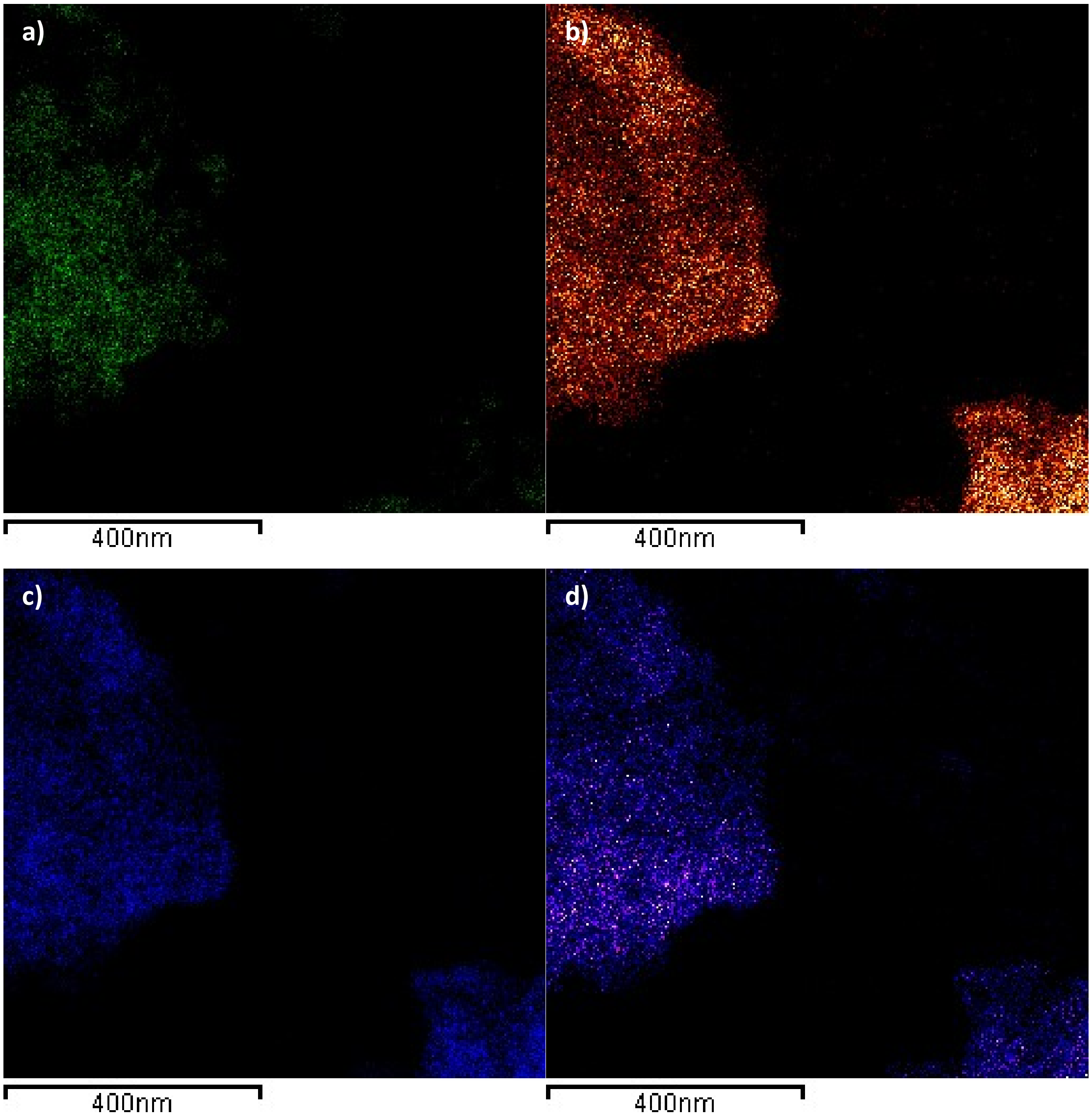

Fig. S20 Chemical mapping of ZnCuFe-LDH from the micrograph on Fig. S18. a) Fe, b) Zn, c) Cu, and d) O.

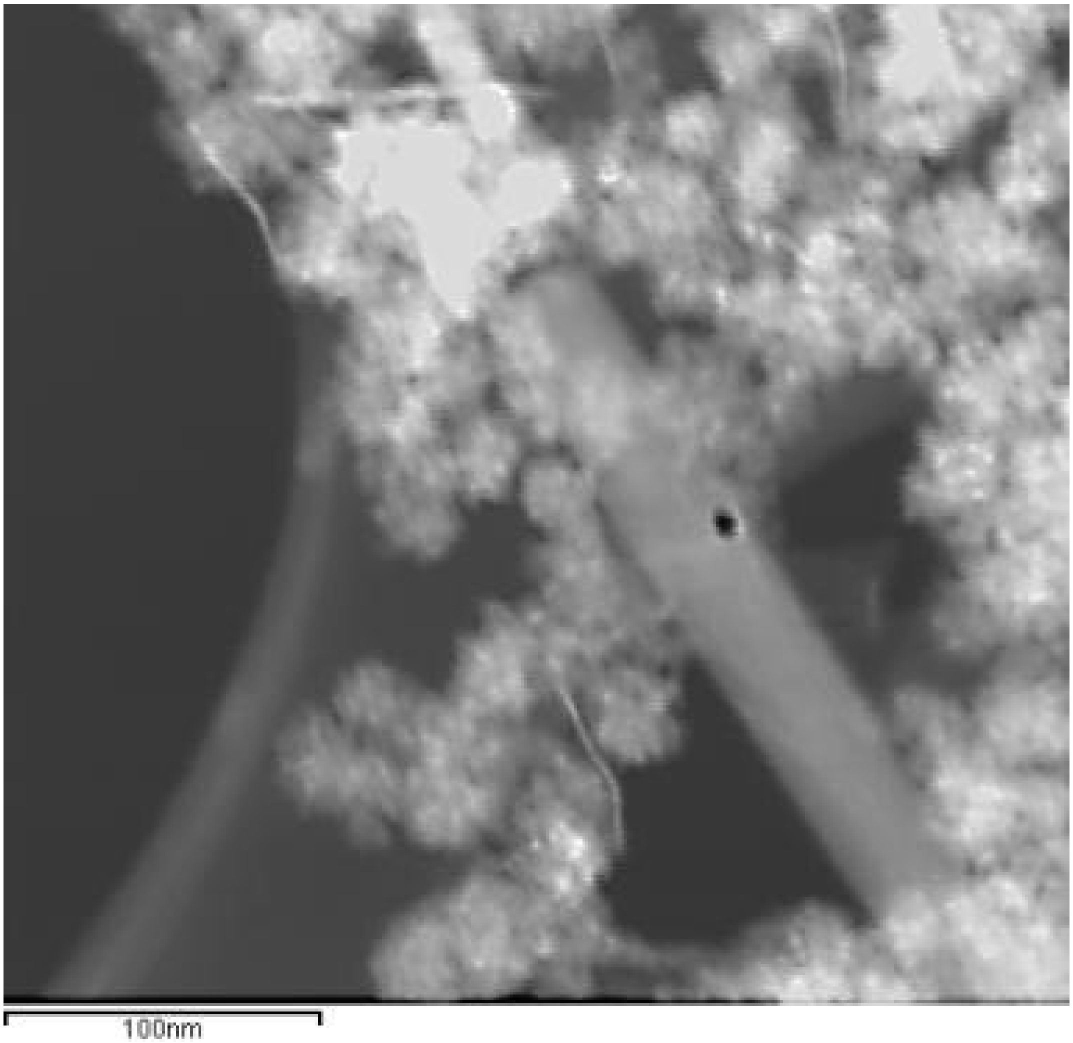

Fig. S21 Dark-field TEM micrograph of CoCuFe-LDH.

Fig. S21 shows a dark field TEM micrograph of the CoCuFe-LDH material and its corresponding chemical mapping. Fig. S22 shows that for this material the nanoparticles seen in the dark-field micrograph are composed of Fe, Co, Cu. Furthermore, it is seen that the nanorod (diagonal rod ~30 nm wide) is composed of Co and Cu as long as it shows a clear absence of Fe. In this case, the Cu mapping shows copper presence throughout the micrograph, due to the grid used is made of copper and generates an overlapping, however, it can be seen that the material is made up of.

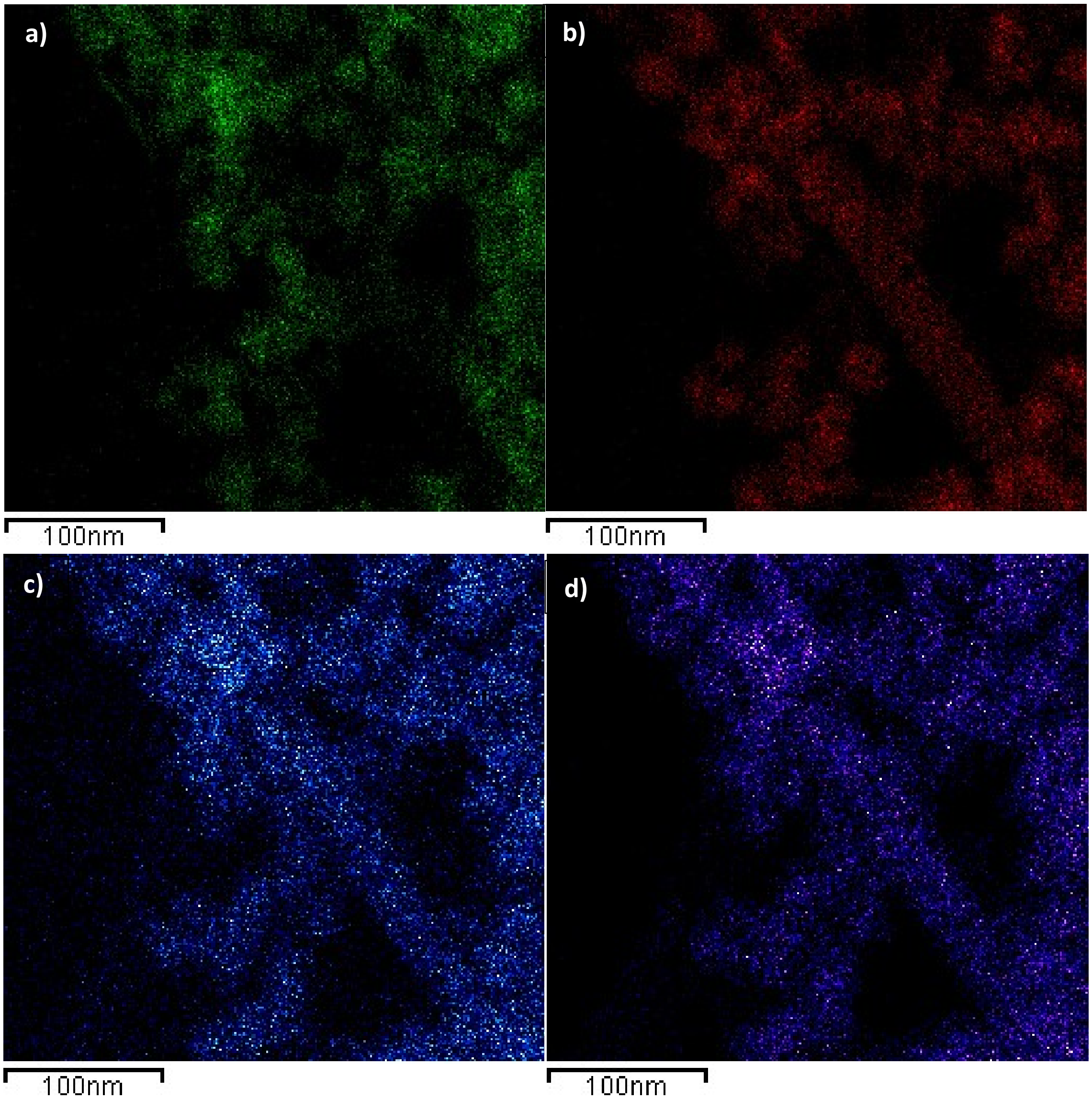

Fig. S22 Chemical mapping of CoCuFe-LDH (Fig. S20). a) Fe, b) Co, c) Cu, and d) O.

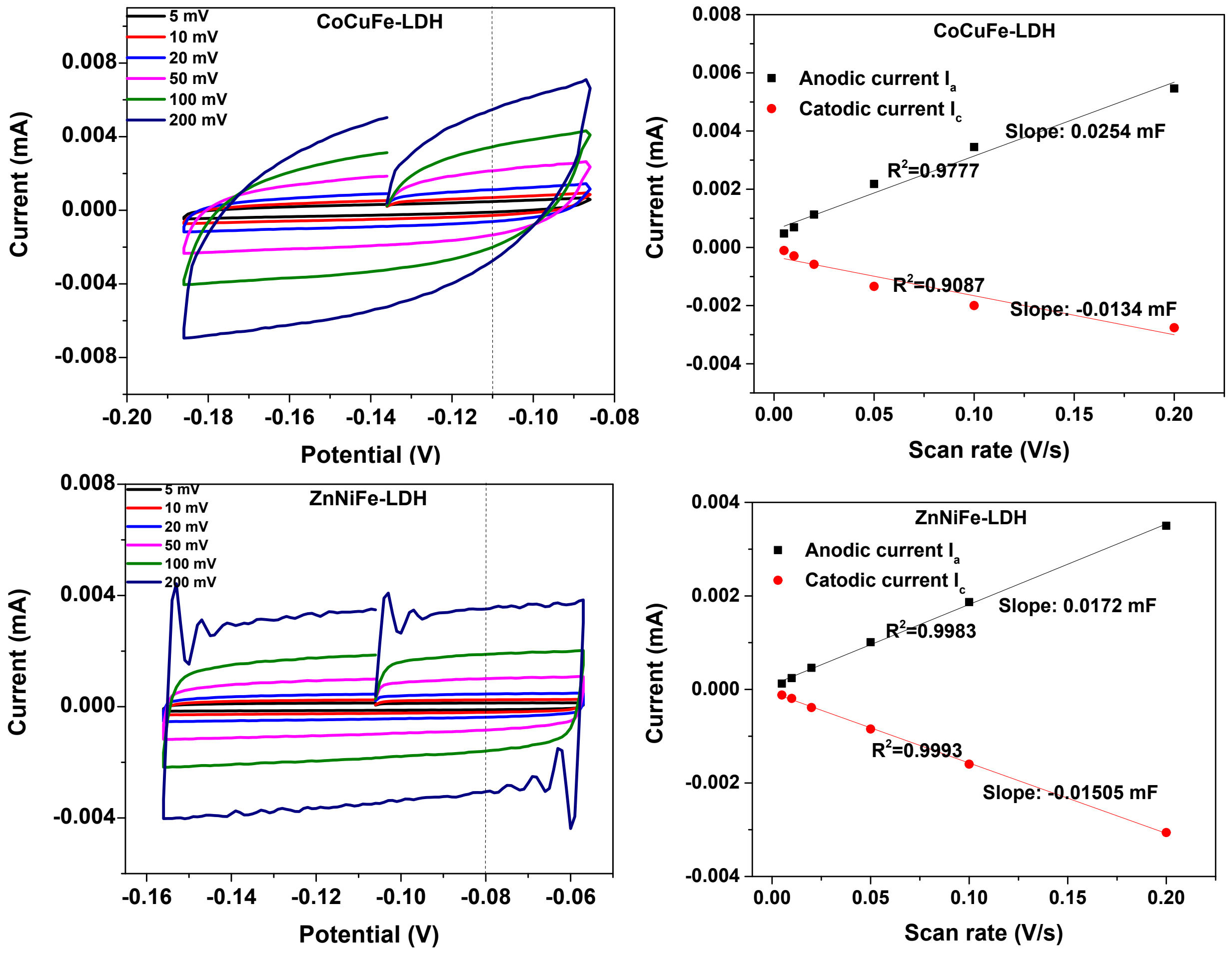

Fig. S23 a) Cyclic voltammogram of CoGaFe-LDH. b) Current *vs.* scan rate plot to determine the electrochemically active surface area for the CoCuFe-LDH catalyst. a) Cyclic voltammogram of ZnNiFe-LDH. b) Current *vs.* scan rate plot to determine the electrochemically active surface area for ZnNiFe-LDH catalyst.

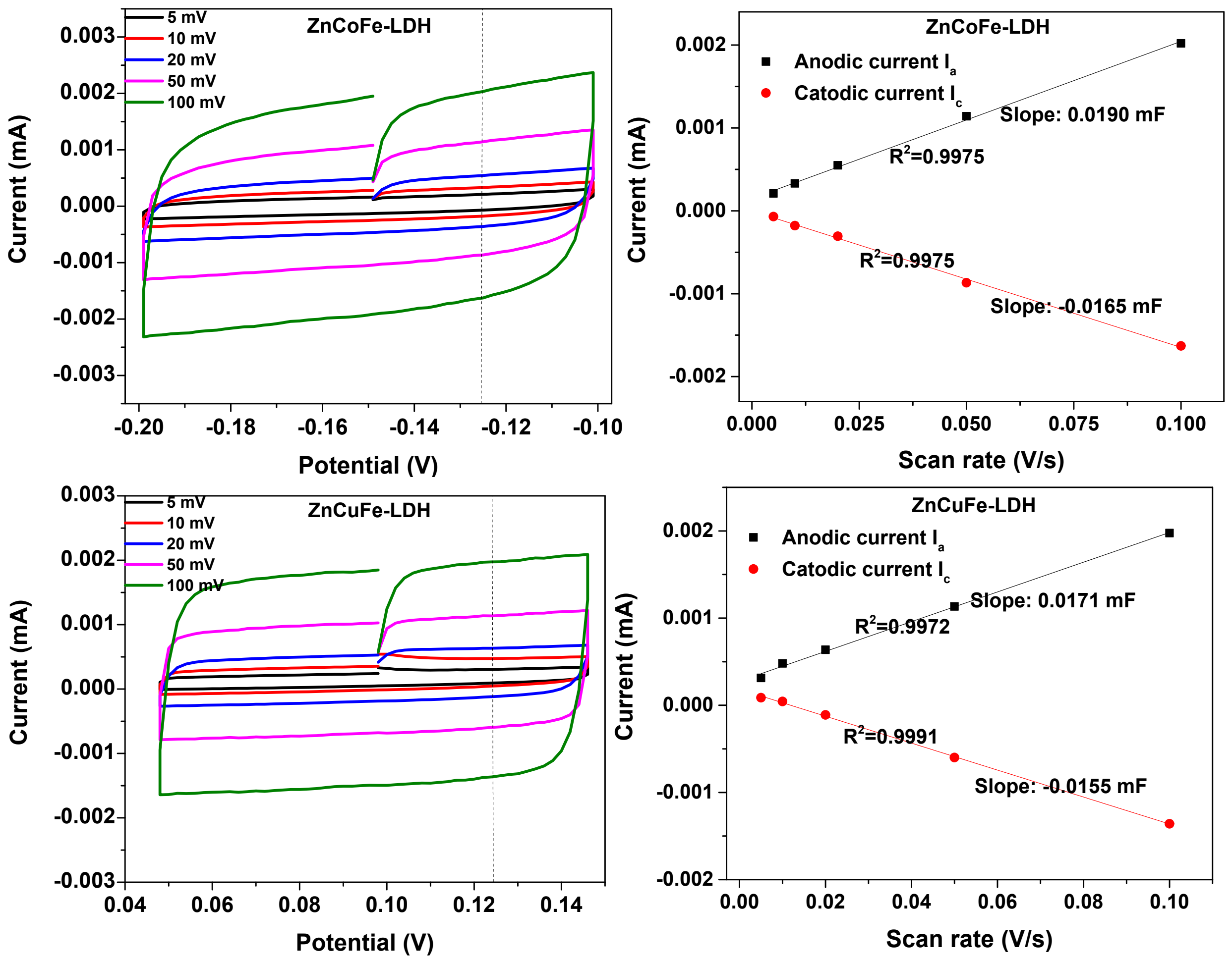

Fig. S24 a) Cyclic voltammogram of ZnCoFe-LDH. b) Current *vs.* scan rate plot to determine the electrochemically active surface area for the ZnCoFe-LDH catalyst. a) Cyclic voltammogram of ZnCuFe-LDH. b) Current *vs.* scan rate plot to determine the electrochemically active surface area for the ZnCuFe-LDH catalyst.

Table S1. Comparison between textural properties of some LDHs.

Material	BET surface area (m^2)	Pore volume (cm^2/g)	Pore diameter (nm)	Preparation method	Refere-nce
MgAl-LDH	6.1	0.017	2.16	Co-precipitation	1
CoAl-LDH	58.6*	0.28	15.40	Co-precipitation	2
ZnAl-LDH	50.0	0.18	11.00	Co-precipitation	3
MgCuAl-LDH	74.9*			Co-precipitation	4
CoNiFe-LDH	91.0			Single-roller melting technique and a dealloying treatment	5

*This is the highest BET surface area for this material, researchers tested several molar ratios and we selected the maximum area.

Table S2. Atomic percentage measured through EDS present in as-synthesized LDHs.

Material	%Ni	%Co	%Ga	%Cu	%Fe	%Zn
CoCuFe-LDH	—	31.96	—	37.73	30.31	—
ZnNiFe-LDH	31.07	—	—	—	29.58	39.35
ZnCoFe-LDH	—	28.88	—	—	39.93	31.89
ZnCuFe-LDH	—	—	—	27.62	34.56	37.82
CoGaFe-LDH	—	71.72	10.37	—	17.91	—

Table S3. Experimental atomic ratio calculate from EDS data, of the as-synthesized LDHs (M=Metal).

Material (M1M2M3-LDH)	Number of M1 atoms	Number of M2 atoms	Number of M3 atoms
CoCuFe-LDH	3	4	3
ZnNiFe-LDH	3	4	3
ZnCoFe-LDH	3	3	4
ZnCuFe-LDH	4	3	4
CoGaFe-LDH	30	3	5

Table S4. Position of photoemission $2p_{3/2}$ peak for all the elements in the as-synthesized LDHs.

Material	Fe (eV)	Co (eV)	Cu (eV)	Zn (eV)	Ni (eV)	Ga (eV)
CoCuFe-LDH	711.4	781.7	935.1	—	—	—
ZnNiFe-LDH	711.6	—	—	1021.4	856.0	—
ZnCoFe-LDH	711.4	781.4	—	1022.1	—	—
ZnCuFe-LDH	711.3	—	935.2	1022.2	—	—
CoGaFe-LDH	—	781.2	—	—	—	1117.5

Table S5. Spin-orbit splitting doublet 2p ($2p_{3/2}$ and $2p_{1/2}$) for all as-synthesized LDHs.

Material	ΔFe (eV)	ΔCo (eV)	ΔCu (eV)	ΔZn (eV)	ΔNi (eV)	ΔGa (eV)
CoCuFe-LDH	13.1	15.5	19.9	—	—	—
ZnNiFe-LDH	13.3	—	—	23.1	17.7	—
ZnCoFe-LDH	13.1	15.5	—	23.1	—	—
ZnCuFe-LDH	13.0	—	19.9	23.1	—	—
CoGaFe-LDH	—	15.7	—	—	—	26.8

Table S6 O_I, O_{II}, O_{III}, and O_{IV} position on the synthesized materials.

Material	O_I	O_{II}	O_{III}	O_{IV}
CoCuFe-LDH	529.5	530.6	532.0	533.7
ZnNiFe-LDH	529.2	530.2	531.6	533.3
ZnCoFe-LDH	529.6	531.3	532.4	533.6
ZnCuFe-LDH	529.5	531.6	532.6	533.7
CoGaFe-LDH	528.7	530.1	531.6	533.2

Table S7. Electrocatalytic comparison between LDHs for water oxidation reaction in alkaline 1 M KOH conditions.

**Catalyst**	**Electrode**	**Overpotential (mV)**	**Current density (mA/cm^2)**	**Tafel slope (mV/dec)**	**Reference**
**CoGaFe-LDH**	**GCE**	**369**	**10**	**64.8**	**This work**
CoCuFe-LDH	GCE	416	10	91.9	This work
ZnCoFe-LDH	GCE	413	10	64.9	This work
ZnNiFe-LDH	GCE	488	10	71.4	This work
CoFe-LDH doped with V	CC	440	10	74	6
CoFe-LDH doped with Cr	NF	238	10	107	7
NiFeMn-LDH	CF	289	20	47	8

GCE: Glassy Carbon Electrode; **NF:** Nickel Foam; **CC:** Carbon Cloth; **CF:** Carbon Fiber.

References:

[1] A. Silva, J. Dias, A. Ferreira and L. F. Brum, A Ternary Catalytic System for the Room Temperature Suzuki-Miyaura Reaction in Water. *The Scientific World Journal*, 2013, 1–8.

[2] M. Huang, X. Wu, X. Yi, G. Han, W. Xia and H. Wan, Highly dispersed CoOx in layered double oxides for oxidative dehydrogenation of propane: guest–host interactions. *RSC Advances*, 2017, *7*(24), 14846–14856.

[3] M. Mureseanu, T. Radu, R. Andrei, M. Darie and G. Carja, Green synthesis of g-C_3N_4/CuONP/LDH composites and derived g-C_3N_4/MMO and their photocatalytic performance for phenol reduction from aqueous solutions. *Applied Clay Science*, 2017, *141*, 1–12.

[4] T-T. Hoang, X-T. Thi, C. Quoc and P. Hoang, Porous metal oxides derived from Cu–Al layered double hydroxide as an efficient heterogeneous catalyst for the Friedel–Crafts alkylation of indoles with benzaldehydes under microwave irradiation. *Heliyon*, 2018, *4*(11), e00966.

[5] C. Dong, L. Han, C. Zhang and Z. Zhang, Scalable Dealloying Route to Mesoporous Ternary CoNiFe Layered Double Hydroxides for Efficient Oxygen Evolution. *ACS Sustainable Chemistry & Engineering*, 2018, *6*(12), 16096–16104.

[6] B. Singh and A. Indra. Tuning the properties of CoFe-layered double hydroxide by vanadium substitution for improved water splitting activity. Dalton Trans., 2021, 50, 2359–2363.

[7] L. Wen, X. Zhang, J. Liu, X. Li, C. Xing, X. Lyu, W. Cai, W. Wang, Y. Li, Cr-Dopant Induced Breaking of Scaling Relations in CoFe Layered Double Hydroxides for Improvement of Oxygen Evolution Reaction, *Small* 15 (2019) 1902373.

[8] Z. Lu, L. Qian, Y. Tian, Y. Li, X. Sun, X. Duan, Ternary NiFeMn Layered Double Hydroxides as High efficient Oxygen Evolution Catalysts, *Chemical Communications.***,** 2016,**52**, 908–911.
